# A CRISPR Interference Platform for Selective Downregulation of Gene Expression in Borrelia burgdorferi

**DOI:** 10.1128/AEM.02519-20

**Published:** 2021-01-29

**Authors:** Constantin N. Takacs, Molly Scott, Yunjie Chang, Zachary A. Kloos, Irnov Irnov, Patricia A. Rosa, Jun Liu, Christine Jacobs-Wagner

**Affiliations:** a Department of Biology and ChEM-H Institute, Stanford University, Stanford, California, USA; b Microbial Sciences Institute, Yale University, West Haven, Connecticut, USA; c Department of Molecular, Cellular, and Developmental Biology, Yale University, New Haven, Connecticut, USA; d Howard Hughes Medical Institute, Stanford University, California, USA; e Department of Microbial Pathogenesis, Yale University School of Medicine, New Haven, Connecticut, USA; f Microbiology Program, Yale University, New Haven, Connecticut, USA; g Laboratory of Bacteriology, Rocky Mountain Laboratories, Division of Intramural Research, National Institute of Allergy and Infectious Diseases, National Institutes of Health, Hamilton, Montana, USA; The Pennsylvania State University

**Keywords:** CRISPR, *Borrelia*, Lyme disease, spirochete, dCas9, cell morphogenesis, bacteria, MreB, RodA, FtsI

## Abstract

The spirochete Borrelia burgdorferi causes Lyme disease, an increasingly prevalent infection. While previous studies have provided important insight into B. burgdorferi biology, many aspects, including basic cellular processes, remain underexplored. To help speed up the discovery process, we adapted a clustered regularly interspaced palindromic repeats interference (CRISPRi) platform for use in B. burgdorferi. For efficiency and flexibility of use, we generated various CRISPRi template constructs that produce different basal and induced levels of *dcas9* and carry different antibiotic resistance markers. We characterized the effectiveness of our CRISPRi platform by targeting the motility and cell morphogenesis genes *flaB*, *mreB*, *rodA*, and *ftsI*, whose native expression levels span 2 orders of magnitude. For all four genes, we obtained gene repression efficiencies of at least 95%. We showed by darkfield microscopy and cryo-electron tomography that flagellin (FlaB) depletion reduced the length and number of periplasmic flagella, which impaired cellular motility and resulted in cell straightening. Depletion of FtsI caused cell filamentation, implicating this protein in cell division in B. burgdorferi. Finally, localized cell bulging in MreB- and RodA-depleted cells matched the locations of new peptidoglycan insertion specific to spirochetes of the Borrelia genus. These results therefore implicate MreB and RodA in the particular mode of cell wall elongation of these bacteria. Collectively, our results demonstrate the efficiency and ease of use of our B. burgdorferi CRISPRi platform, which should facilitate future genetic studies of this important pathogen.

**IMPORTANCE** Gene function studies are facilitated by the availability of rapid and easy-to-use genetic tools. Homologous recombination-based methods traditionally used to genetically investigate gene function remain cumbersome to perform in B. burgdorferi, as they often are relatively inefficient. In comparison, our CRISPRi platform offers an easy and fast method to implement, as it only requires a single plasmid transformation step and isopropyl-β-d-thiogalactopyranoside (IPTG) addition to obtain potent (>95%) downregulation of gene expression. To facilitate studies of various genes in wild-type and genetically modified strains, we provide more than 30 CRISPRi plasmids that produce distinct levels of *dcas9* expression and carry different antibiotic resistance markers. Our CRISPRi platform represents a useful and efficient complement to traditional genetic and chemical methods to study gene function in B. burgdorferi.

## Abstract (precis)

Gene function studies are facilitated by the availability of rapid and easy-to-use genetic tools. Homologous recombination-based methods traditionally used to genetically investigate gene function remain cumbersome to perform in B. burgdorferi, as they often are relatively inefficient.

## INTRODUCTION

Borrelia burgdorferi, a spirochete, is maintained in nature via a transmission cycle between a tick vector and a warm-blooded host, such as a wild mouse (1). A bite by a B. burgdorferi-colonized tick can lead to transmission of the bacterium to humans. In the absence of timely antibiotic treatment, infection by B. burgdorferi causes Lyme disease, a prevalent vector-borne disease in temperate regions of the Northern Hemisphere (2). The infection can result in a wide variety of symptoms, ranging from malaise, fever, and a characteristic skin rash during early stages of the disease, to cardiac, neurologic, or articular pathologies in later stages (3). The rapid rise in Lyme disease incidence in recent years (2) underscores the need for a detailed understanding of not only the disease, but also the pathogen itself.

Studying the biology of a pathogen is greatly facilitated by the genetic tractability of the organism. For this reason, a diverse set of genetic reagents and techniques have been developed and validated for use in B. burgdorferi (4–6). However, creating gene deletion mutants in B. burgdorferi remains tedious and time consuming. Homologous recombination of suicide vectors is needed to create gene deletion mutants. In B. burgdorferi, this process occurs at frequencies of ∼10^−7^ (7–9), which are close to the 10^−9^ to 10^−7^ range of frequencies at which spontaneous mutants conferring resistance to common selection antibiotics arise (10). This inefficient process is compounded by B. burgdorferi’s slow *in vitro* growth rate, with typical doubling times in the range of 5 to 18 h (11–16). Genetic modification is particularly challenging for genes that are essential for viability; such genes cannot be deleted, and generation of conditional mutants (17) often requires multiple transformation steps and is therefore even slower and less efficient.

**FIG 1:**
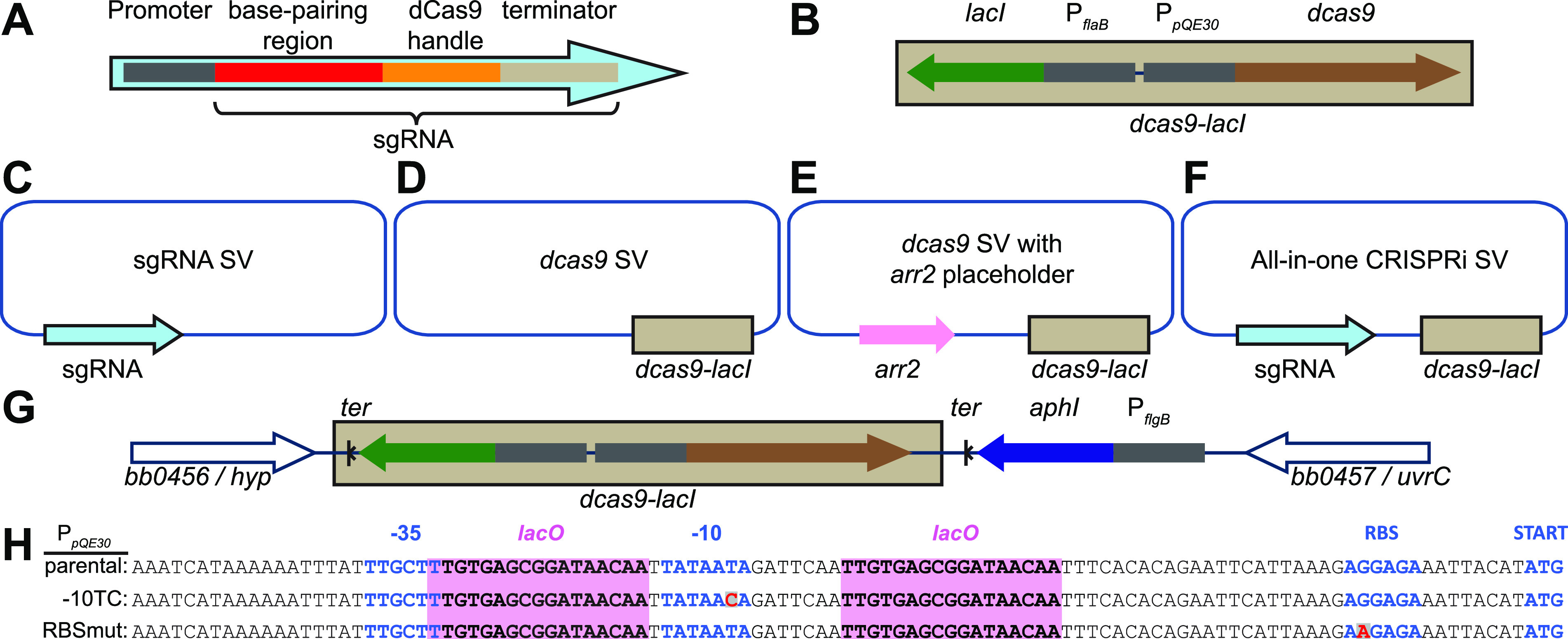
Summary of relevant genetic constructs. (A) Schematic of a mature short guide RNA (sgRNA) cassette, including its promoter and relevant functional parts. (B) Schematic of the inducible *dcas9-lacI* cassette. (C to F) Schematic of shuttle vectors (SV) generated and used in this study. The Borrelia burgdorferi and Escherichia coli origins of replication and the antibiotic resistance markers are not shown. *arr2*, rifampin resistance cassette. (G) Schematic of the chromosomally encoded *dcas9* locus of strain CJW_Bb362. The *dcas9-lacI* cassette and a kanamycin resistance cassette were inserted in the intergenic region between genes *bb0456* and *bb0457*. *ter*, transcriptional terminator; *aphI*, kanamycin resistance gene. (H) Mutations introduced into the P*_pQE30_* sequence that successfully decreased basal expression of *dcas9*. *lacO*, LacI binding sites; RBS, ribosomal binding site. Mutated residues are shown in red.

The development of clustered regularly interspaced palindromic repeats interference (CRISPRi) (18, 19) provides an attractive complement to traditional genetic manipulation protocols based on homologous recombination. CRISPRi is a gene product depletion method that requires two components. One of them is dCas9, a catalytically inactive version of Cas9, which is the nuclease component of a bacterial adaptive immunity system against invading DNA molecules (18, 20, 21). The other component is a short guide RNA molecule, or sgRNA. The sgRNA (Fig. 1A) contains a base-pairing region and a dCas9 recognition loop, which is called a dCas9 handle (18, 20). The base-paring region is usually a 20-nucleotide stretch complementary to the target DNA strand (18, 19). Proper targeting also requires a protospacer adjacent motif (PAM), located in the DNA next to the base-pairing region. When coexpressed, dCas9 and the sgRNA form a complex that scans the DNA until it finds a PAM-proximal sequence complementary to the sgRNA’s base-pairing region (22). There, the dCas9-sgRNA complex stably binds to the DNA. When dCas9 is targeted to a promoter or operator, it blocks transcription initiation. When dCas9 is targeted to the coding sequence or the 5′ untranslated region (5′ UTR) of a gene, it blocks transcription elongation (18). Together, the required sgRNA-DNA complementary base pairing and presence of an adjacent PAM site render CRISPRi highly specific (18). Since its development, the CRISPRi method has been adapted for the study of a wide variety of organisms and cell types, from mammalian cells and yeast (23) to various bacterial species, including Escherichia coli (18), Bacillus subtilis (24, 25), Caulobacter crescentus (26, 27), Mycobacterium tuberculosis (28–30), and Leptospira interrogans (31), to name just a few.

Here, we have adapted the CRISPRi system for use in B. burgdorferi. We present multiple versions of the platform that are characterized by efficient gene product downregulation, ease of use, and relatively fast clone generation. We have evaluated the functionality of these versions by targeting genes that have broadly varied native expression levels and are involved in B. burgdorferi motility, cell shape determination, and cell division.

## RESULTS

### Constructs and strains for sgRNA and *dcas9* expression in B. burgdorferi.

Based on genome sequence analysis, B. burgdorferi does not encode an endogenous Cas9 protein. To adapt the Streptococcus pyogenes-derived CRISPRi system (18, 19) for use in B. burgdorferi, we assembled expression cassettes for sgRNAs and *dcas9* (Fig. 1A and B). The mature sgRNA cassettes that we designed contain the sgRNA sequence fused to the transcriptional start site of a constitutive promoter (Fig. 1A). To generate mature sgRNA cassettes, we first assembled “template” cassettes, which contain the promoter driving sgRNA expression, an ∼500-bp DNA filler sequence derived from a firefly luciferase gene (32) and the sgRNA’s dCas9 handle (see Fig. S1A in the supplemental material). We then released the DNA filler sequence by digestion with SapI (or its isoschiomers BspQI or LguRI) and ligated in its place a pair of annealed primers (Fig. S1A and B). We designed these primers such that they encode the sgRNA’s base-pairing region (see Materials and Methods). For sgRNA expression, we primarily employed a synthetic promoter, J23119, here referred to as P*_syn_*, which was previously used to drive sgRNA expression in E. coli (18, 19). Using a transcriptional fusion to mCherry, we showed that P_syn_ is active in B. burgdorferi (Fig. S1C and D). To drive sgRNA expression, we also tested multiple B. burgdorferi promoters (see Table S1 in the supplemental material) whose strengths we previously characterized (Fig. S1D) (6). The nature of the promoter driving sgRNA expression, however, did not appear to affect the functionality of the CRISPRi platform (see below). We generated both the template and the mature sgRNA cassettes in B. burgdorferi/E. coli shuttle vectors and refer to them as sgRNA shuttle vectors (Fig. 1C and Fig. S1A and Table 1).

**TABLE 1:** Plasmids used or generated in this study^*a*^

Plasmid name	Relevant notes	Antibiotic resistance^*b*^	Source or reference [Addgene reference no.]^*c*^
Plasmids previously published			
Sources for *dcas9* and the inducible system			
pdCas9-bacteria	Source of *dcas9*	Cm	18 [44249]
pJSB252	Backbone for *dcas9* vector	Sm, Sp	32
Empty shuttle vectors			
pBSV2G		Gm	8
pBSV2G_2		Gm	6 [118225]
pBSV2		Km	78
pBSV2_2		Km	6 [118226]
pKFSS1_2		Sm, Sp	6 [118227]
pBSV2B		Bs, Rf	6 [118228]
pBSV2H		Hy, Rf	6 [118229]
Shuttle vectors carrying promoter and fluorescent protein sequences			
pBSV2G_P_flaB_-msfGFP^Bb^		Gm	6 [118231]
pBSV2_P_resT_-mCherry^Bb^		Km	6 [118238]
pBSV2_P_0026_-mCherry^Bb^		Km	6 [118239]
pBSV2_P_0031_-mCherry^Bb^		Km	6 [118240]
pBSV2_P_0526_-mCherry^Bb^		Km	6 [118241]
pBSV2_P_0826_-mCherry^Bb^		Km	6 [118242]
Plasmids generated in this study			
Hygromycin B-resistant shuttle vector with a single copy of the antibiotic resistance cassette			
pBSV2H_2		Hy, Rf	
P_syn_-mCherry^Bb^ reporter shuttle vector			
pBSV2_P_syn_-mCherry^Bb^		Km	[149636]
sgRNA shuttle vectors carrying a template sgRNA500 cassette			
pBSV2G_P_syn_-sgRNA500		Gm	[149614]
pBSV2G_P_flaBS_-sgRNA500		Gm	[149615]
pBSV2G_P_resTS_-sgRNA500		Gm	[149616]
pBSV2G_P_resTL_-sgRNA500		Gm	[149617]
pBSV2G_P_0826S_-sgRNA500		Gm	[149618]
pBSV2G_P_0826L_-sgRNA500		Gm	[149619]
pBSV2G_P_0026_-sgRNA500		Gm	[149620]
pBSV2G_P_0526_-sgRNA500		Gm	[149621]
pBSV2G_P_0031_-sgRNA500		Gm	[149622]
pBSV2_P_syn_-sgRNA500		Km	[149613]
pKFSS1_P_syn_-sgRNA500		Sm, Sp	[149557]
pBSV2B_P_syn_-sgRNA500		Bs, Rf	[149558]
pBSV2H_P_syn_-sgRNA500^*d*^		Hy, Rf	[149559]
sgRNA shuttle vectors carrying mature sgRNA cassettes targeting *flaB*, *ftsI*, *mreB*, or *rodA*			
pBSV2G_P_syn_-sgRNAflaB		Gm	[149560]
pBSV2G_P_flaBS_-sgRNAflaB		Gm	[149623]
pBSV2G_P_resTS_-sgRNAflaB		Gm	[149624]
pBSV2G_P_resTL_-sgRNAflaB		Gm	[149625]
pBSV2G_P_0826S_-sgRNAflaB		Gm	[149626]
pBSV2G_P_0826L_-sgRNAflaB		Gm	[149627]
pBSV2G_P_0026_-sgRNAflaB		Gm	[149628]
pBSV2G_P_0526_-sgRNAflaB		Gm	[149629]
pBSV2G_P_0031_-sgRNAflaB		Gm	[149630]
pBSV2_P_syn_-sgRNAflaB		Km	[149631]
pBSV2H_P_syn_-sgRNAflaB		Hy, Rf	[149561]
pBSV2G_P_syn_-sgRNAftsI		Gm	[149562]
pBSV2_P_syn_-sgRNAftsI		Km	[149632]
pBSV2H_P_syn_-sgRNAftsI		Hy, Rf	[149563]
pBSV2G_P_syn_-sgRNAmreB		Gm	[149566]
pBSV2_P_syn_-sgRNAmreB		Km	[149634]
pBSV2H_P_syn_-sgRNAmreB		Hy, Rf	[149567]
pBSV2G_P_syn_-sgRNArodA		Gm	[149568]
pBSV2_P_syn_-sgRNArodA		Km	[149635]
pBSV2H_P_syn_-sgRNArodA		Hy, Rf	[149569]
*dcas9* shuttle vectors
pBbdCas9S^*e*^		Sm, Sp	[149638]
pBbdCas9S(RBSmut)		Sm, Sp	[149574]
pBbdCas9S(−10TC)		Sm, Sp	[149581]
pBbdCas9S_arr2		Sm, Sp, Rf	[149639]
pBbdCas9S(RBSmut)_arr2		Sm, Sp, Rf	[149575]
pBbdCas9S(−10TC)_arr2		Sm, Sp, Rf	[149582]
pBbdCas9K_arr2		Km, Rf	[149570]
pBbdCas9K(RBSmut)_arr2		Km, Rf	[149577]
pBbdCas9K(−10TC)_arr2		Km, Rf	[149584]
pBbdCas9G_arr2		Gm, Rf	[149571]
pBbdCas9G(RBSmut)_arr2		Gm, Rf	[149578]
pBbdCas9G(−10TC)_arr2		Gm, Rf	[149585]
pBbdCas9B_arr2		Bs, Rf	[149572]
pBbdCas9B(RBSmut)_arr2		Bs, Rf	[149579]
pBbdCas9B(−10TC)_arr2		Bs, Rf	[149586]
pBbdCas9H_arr2		Hy, Rf	[149573]
pBbdCas9H(RBSmut)_arr2		Hy, Rf	[149580]
pBbdCas9H(−10TC)_arr2		Hy, Rf	[149587]
All-in-one CRISPRi shuttle vectors carrying a template sgRNA500 cassette			
pBbdCas9S_P_syn_-sgRNA500		Sm, Sp	[149640]
pBbdCas9S_P_0526_-sgRNA500		Sm, Sp	[149641]
pBbdCas9S(RBSmut)_P_syn_-sgRNA500		Sm, Sp	[149576]
pBbdCas9S(−10TC)_P_syn_-sgRNA500		Sm, Sp	[149583]
All-in-one CRISPRi shuttle vectors carrying mature sgRNA cassettes targeting *flaB*, *ftsI*, *mreB*, or *rodA*			
pBbdCas9S_P_syn_-sgRNAflaB		Sm, Sp	[149642]
pBbdCas9S_P_flaBS_-sgRNAflaB		Sm, Sp	[149643]
pBbdCas9S_P_resTS_-sgRNAflaB		Sm, Sp	[149644]
pBbdCas9S_P_resTL_-sgRNAflaB		Sm, Sp	[149645]
pBbdCas9S_P_0026_-sgRNAflaB		Sm, Sp	[149646]
pBbdCas9S_P_0826S_-sgRNAflaB		Sm, Sp	[149647]
pBbdCas9S_P_0826L_-sgRNAflaB		Sm, Sp	[149648]
pBbdCas9S(RBSmut)_P_syn_-sgRNAflaB		Sm, Sp	[149588]
pBbdCas9S(−10TC)_P_syn_-sgRNAflaB		Sm, Sp	[149589]
pBbdCas9S_P_syn_-sgRNAftsI		Sm, Sp	[149649]
pBbdCas9S_P_0526_-sgRNAftsI		Sm, Sp	[149650]
pBbdCas9S(RBSmut)_P_syn_-sgRNAftsI		Sm, Sp	[149590]
pBbdCas9S(−10TC)_P_syn_-sgRNAftsI		Sm, Sp	[149591]
pBbdCas9S_P_syn_-sgRNAmreB		Sm, Sp	[149653]
pBbdCas9S_P_0526_-sgRNAmreB		Sm, Sp	[149654]
pBbdCas9S(RBSmut)_P_syn_-sgRNAmreB		Sm, Sp	[149594]
pBbdCas9S(−10TC)_P_syn_-sgRNAmreB		Sm, Sp	[149595]
pBbdCas9S_P_syn_-sgRNArodA		Sm, Sp	[149655]
pBbdCas9S_P_0526_-sgRNArodA		Sm, Sp	[149658]
pBbdCas9S(RBSmut)_P_syn_-sgRNArodA		Sm, Sp	[149656]
pBbdCas9S(−10TC)_P_syn_-sgRNArodA		Sm, Sp	[149657]
pBbdCas9S(−10AC1)_P_syn_-sgRNArodA		Sm, Sp	[149658]
pBbdCas9S(−10AC2)_P_syn_-sgRNArodA		Sm, Sp	[149660]
pBbdCas9S(−10AC12)_P_syn_-sgRNArodA		Sm, Sp	[149661]
Suicide vector for chromosomal integration of the *dcas9-lacI* cassette			
pKIKan_idCas9_Chr_center		Km	[149637]

a This table lists the plasmids used or generated in this study. As indicated, these plasmids are available to the scientific community through Addgene. This includes plasmids that we generated but did not specifically test. For example, for the chromosomal *dcas9* variation of the CRISPR interference (CRISPRi) platform, our characterization used the pBSV2H-based series of sgRNA shuttle vectors. However, we also generated pBSV2- and pBSV2G-based series. We do not expect their behavior to be different from that of the series we tested. We therefore provide these plasmids along with the characterized versions in the hope that they will be useful to others.

b Cm, chloramphenicol; Gm, gentamicin; Km, kanamycin; Sm, streptomycin; Sp, spectinomycin; Bs, blasticidin S; Rf, rifampin; Hy, hygromycin B.

c Addgene record numbers are given within brackets for plasmids obtained by us from Addgene, previously generated by us (6) and available from Addgene, or generated by us as part of this study and deposited at Addgene.

d The 500-bp filler of the sgRNA500 template cassette of vector pBSV2H_P_syn_-sgRNA500 cannot be replaced with the base-pairing region of a mature sgRNA by SapI digestion followed by ligation of annealed primers (see Fig. S1A and B in the supplemental material) because the hygromycin resistance gene *hph^Bb^* also contains a SapI site. Instead, site-directed mutagenesis, Gibson assembly, or transfer of a mature sgRNA cassette from a different sgRNA shuttle vector can be used to generate a hygromycin B-resistant, mature sgRNA-carrying shuttle vector.

e The name pBbdCas9X stands for inducible expression of *dcas9* in Borrelia burgdorferi. The letter X (with X = S, K, G, H, or B) denotes the antibiotic resistance for B. burgdorferi selection (streptomycin, kanamycin, gentamicin, hygromycin B, or blasticidin S, respectively).

To express *dcas9*, we generated a *dcas9*-*lacI* cassette (Fig. 1B), which contains the *dcas9* gene controlled by the isopropyl-β-d-thiogalactopyranoside (IPTG)-inducible P*_pQE30_* promoter and a constitutively expressed *lacI* gene, both derived from plasmid pJSB252 (32). When the *dcas9*-*lacI* cassette is expressed from a B. burgdorferi shuttle vector, we here refer to such a vector as a *dcas9* shuttle vector (Fig. 1D and Table 1). To facilitate cloning of a sgRNA cassette into the *dcas9* shuttle vector, we generated cloning intermediates that contain a rifampin resistance cassette placeholder (Fig. 1E and Fig. S1E and Table 1). Replacement of the rifampin cassette with sgRNA cassettes yielded all-in-one CRISPRi shuttle vectors (Fig. 1F and Fig. S1E and Table 1). To facilitate use of the CRISPRi platform in a variety of B. burgdorferi strains, including ones that already contain antibiotic resistance markers from prior genetic modifications, we generated five variations of the *dcas9* shuttle vectors, each carrying a different antibiotic resistance marker (Table 1).

We also inserted the *dcas9-lacI* cassette into an intergenic region of the chromosome of the infectious, transformable, B31-derived B. burgdorferi strain B31-A3-68 Δ*bbe02*::*P_flaB_-aadA* (33), generating strain CJW_Bb362 (Fig. 1G and Table 2). This strain allows for stable maintenance of the *dcas9-lacI* cassette in the absence of antibiotic selection. It requires transformation with a sgRNA shuttle vector (Fig. 1C) to generate a CRISPRi strain for protein depletion, while transformation with an empty shuttle vector yields a control strain.

**TABLE 2:** B. burgdorferi strains used or generated in this study

Strain	Genotype/description^*a*^	Antibiotic resistance^*b*^	Source or reference
Strains previously published			
B31 e2	Reduced genome derivative of the type strain B31	None	75
B31-A3-68-Δ*bbe02*::*P_flaB_*-*aadA*	Transformable infectious derivative of the type strain B31; genotype B31-A3-68 lp25[Δ*bbe02*::P*_flaB_*-*aadA*] cp9^−^ lp5^−^ lp56^−^; strain routinely referred to as “S9”	Sm	33
B31-A3-68-Δ*bbe02*::*P_flgB_*-*aphI*	Transformable infectious derivative of the type strain B31; genotype B31-A3-68 lp25[Δ*bbe02*::P*_flgB_*-*aphI*] cp9^−^ lp5^−^ lp56^−^; strain routinely referred to as “K2”	Km	33
Strains generated as part of this study			
P*_syn_*-mCherry fluorescence reporter strain			
CJW_Bb122	B31 e2 / pBSV2_P_syn_-mCherry^Bb^	Km	
Chromosomal insertion of the *dcas9-lacI* cassette			
CJW_Bb362	Genotype B31-A3-68 Chr[Δnt476219-476250::(P*_flgB_-aphI*_P*_flaB_-lacI*_P*_pQE30_-dcas9*)] lp25[Δ*bbe02*::P*_flaB_*-*aadA*] cp9^−^ lp5^−^ lp56^−^	Km, Sm	[NR-53512]^*c*^
Control CRISPRi strains (expressing no sgRNA cassette)			
CJW_Bb242	K2/pBbdCas9S	Km, Sm	
CJW_Bb410	K2/pBbdCas9S(RBSmut)	Km, Sm	
CJW_Bb411	K2/pBbdCas9S(−10TC)	Km, Sm	
CJW_Bb430	CJW_Bb362/pBSV2H	Km, Sm, Hy	
*flaB* CRISPRi strains			
CJW_Bb228	K2/pBbdCas9S_P_resTL_-sgRNAflaB	Km, Sm	
CJW_Bb234	K2/pBbdCas9S_P_0826L_-sgRNAflaB	Km, Sm	
CJW_Bb235	K2/pBbdCas9S_P_0826S_-sgRNAflaB	Km, Sm	
CJW_Bb290	K2/pBbdCas9S_P_resTS_-sgRNAflaB	Km, Sm	
CJW_Bb312	K2/pBbdCas9S_P_flaBS_-sgRNAflaB	Km, Sm	
CJW_Bb313	K2/pBbdCas9S_P_syn_-sgRNAflaB	Km, Sm	
CJW_Bb314	K2/pBbdCas9S_P_0026_-sgRNAflaB	Km, Sm	
CJW_Bb381	K2/pBbdCas9S(RBSmut)_P_syn_-sgRNAflaB	Km, Sm	
CJW_Bb385	K2/pBbdCas9S(−10TC)_P_syn_-sgRNAflaB	Km, Sm	
CJW_Bb404	CJW_Bb362/pBSV2H_P_syn_-sgRNAflaB	Km, Sm, Hy	
*ftsI* CRISPRi strains			
CJW_Bb351	K2/pBbdCas9S_P_syn_-sgRNAftsI	Km, Sm	
CJW_Bb363	K2/pBbdCas9S_P_0526_-sgRNAftsI	Km, Sm	
CJW_Bb383	K2/pBbdCas9S(RBSmut)_P_syn_-sgRNAftsI	Km, Sm	
CJW_Bb386	K2/pBbdCas9S(−10TC)_P_syn_-sgRNAftsI	Km, Sm	
CJW_Bb405	CJW_Bb362/pBSV2H_P_syn_-sgRNAftsI	Km, Sm, Hy	
*mreB* CRISPRi strains			
CJW_Bb382	K2/pBbdCas9S(RBSmut)_P_syn_-sgRNAmreB	Km, Sm	
CJW_Bb398	K2/pBbdCas9S(−10TC)_P_syn_-sgRNAmreB	Km, Sm	
CJW_Bb407	CJW_Bb362/pBSV2H_P_syn_-sgRNAmreB	Km, Sm, Hy	
*rodA* CRISPRi strains			
CJW_Bb346	K2/pBbdCas9S_P_syn_-sgRNArodA	Km, Sm	
CJW_Bb367	K2/pBbdCas9S(−10AC12)_P_syn_-sgRNArodA	Km, Sm	
CJW_Bb368	K2/pBbdCas9S(RBSmut)_P_syn_-sgRNArodA	Km, Sm	
CJW_Bb369	K2/pBbdCas9S(−10TC)_P_syn_-sgRNArodA	Km, Sm	
CJW_Bb375	K2/pBbdCas9S(−10AC1)_P_syn_-sgRNArodA	Km, Sm	
CJW_Bb376	K2/pBbdCas9S(−10AC2)_P_syn_-sgRNArodA	Km, Sm	
CJW_Bb380	K2/pBbdCas9S_P_0526_-sgRNArodA	Km, Sm	
CJW_Bb408	CJW_Bb362/pBSV2H_P_syn_-sgRNArodA	Km, Sm, Hy	

a For the two B31-A3-68-Δ*bbe02* strains, K2 and S9, and for CJW_Bb362, a detailed genetic description of relevant genetic elements is provided. This includes the nature of the Δ*bbe02* mutation, the insertion of the *dcas9-lacI* cassette into the chromosome (Chr) and a list of the native B31 plasmids missing from these strains.

b Km, kanamycin; Sm, streptomycin; Hy, hygromycin B.

c Strain CJW_Bb362 was deposited with ATCC and will be available from BEI Resources. Its BEI Resources record number is provided in brackets.

Regardless of the CRISPRi version used (all-in-one CRISPRi shuttle vector or strain CJW_Bb362 transformed with an sgRNA shuttle vector), in the absence of IPTG (uninduced condition), cells of B. burgdorferi CRISPRi strains had their *dcas9* expression repressed by the binding of LacI to the *lacO* sites within the P*_pQE30_* promoter (Fig. 1H). IPTG addition to the cultures (induced condition) releases LacI from P*_pQE30_*, derepresses transcription from this promoter, and leads to synthesis of dCas9. Nonetheless, some background *dcas9* expression occurred in the absence of IPTG. As discussed below, this proved problematic when targeting certain B. burgdorferi genes. To decrease this basal *dcas9* expression, we mutated either the −10 promoter region of P*_pQE30_* or the ribosome-binding site upstream of the *dcas9* translational start codon in the background of the *dcas9* or the all-in-one CRISPRi shuttle vectors (Fig. 1H, Fig. S1F, and Table 1). One of the promoter mutations (−10TC), as well as the ribosome-binding site mutation (RBSmut), proved effective in reducing background expression of *dcas9*, and both were analyzed in greater detail (see below).

Altogether, we generated and characterized four versions of the B. burgdorferi CRISPRi system. One is based on chromosomal expression of *dcas9* paired with plasmid-based expression of the sgRNA. The others are all-in-one plasmid-based versions that express both *dcas9* and the sgRNA from the same plasmid. The plasmid-based versions differ in whether the promoter that controls *dcas9* expression carries no mutation, a promoter mutation (−10TC), or the ribosomal binding site mutation (RBSmut).

### Inducible expression of *dcas9* in B. burgdorferi.

**FIG 2:**
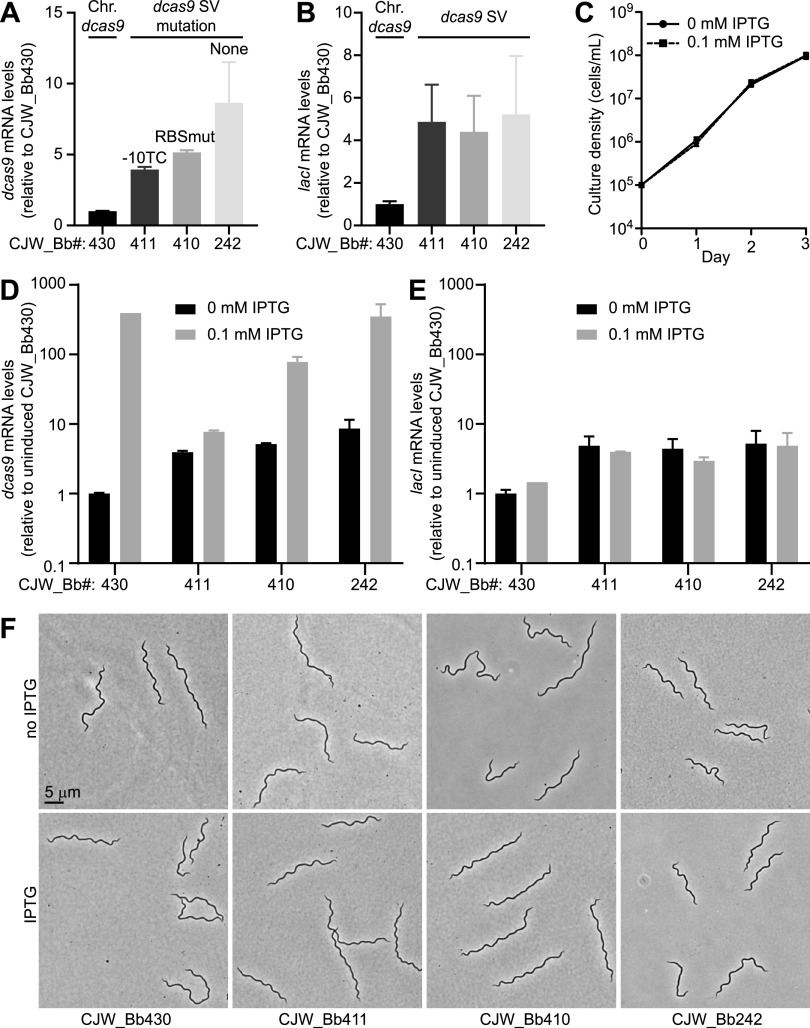
Characterization of *dcas9* expression in B. burgdorferi. (A) Comparison of *dcas9* mRNA levels measured in the absence of isopropyl-β-d-thiogalactopyranoside (IPTG) in control strains that lack an sgRNA. Strain numbers are shown at the bottom. Relevant strain characteristics are marked on the graph. mRNA levels (measured by quantitative real-time PCR [qRT-PCR]) are shown relative to those in strain CJW_Bb430. Chr., chromosomal; SV, shuttle vector. (B) Comparison of *lacI* mRNA levels measured in the absence of IPTG in the same samples as in panel A. (C) Growth curve of strain CJW_Bb242 in the presence of 0 or 0.1 mM IPTG. Cell densities of three replicate cultures were counted daily. Shown are means ± standard deviations. (D) IPTG-mediated induction of *dcas9* expression measured by qRT-PCR in the strains indicated below the graph. All values are reported relative to the levels in the uninduced strain CJW_Bb430. Measurements were obtained after 1 day of induction. (E) *lacI* mRNA levels measured in the same samples as in panel D. (A, B, D, and E) Shown are means ± standard deviations measured from two cultures. A single sample was measured for the induced CJW_Bb430 condition. (F) Phase-contrast images of cells of the indicated strains after 2 days of growth in the presence or absence of IPTG.

To characterize *dcas9* expression in each of the four versions of our B. burgdorferi CRISPRi platform, we generated four corresponding sgRNA-free control strains (Table 2). We first measured, by quantitative real-time PCR (qRT-PCR), their basal *dcas9* expression in the absence of IPTG induction. As expected, all control strains (CJW_Bb242, CJW_Bb410, and CJW_Bb411) harboring *dcas9* on a shuttle vector had higher levels of basal *dcas9* expression than the control strain (CJW_Bb430) carrying a chromosomal *dcas9* (Fig. 2A). Expression of *lacI* was also about 5-fold higher in these strains than in the chromosomal *dcas9* strain (Fig. 2B). These levels could reflect copy number differences between the shuttle vector and the chromosome, previously reported to be in a ratio of about 5:1 (34–36). They could also reflect changes in DNA topology and genomic context that are known to affect gene expression in B. burgdorferi (36–38).

Basal levels of *dcas9* expression varied among the control strains carrying the *dcas9-lacI* cassette on a shuttle vector, with the highest level found in strain CJW_Bb242 (Fig. 2A), which has *dcas9* expression controlled by the parental P*_pQE30_* promoter (Fig. 1H). Mutation of the −10 region of this promoter yielded the largest drop in basal *dcas9* expression (Fig. 2A), likely reflecting lower promoter activity. The RBS mutation found in strain CJW_Bb410 also decreased basal *dcas9* expression levels (Fig. 2A), presumably reflecting lower stability of the *dcas9* mRNA due to lower ribosome occupancy. Translating ribosomes are known to protect bacterial mRNAs from degradation (39). The similar levels of *lacI* expression among the three strains that carry the *dcas9-lacI* cassette on a shuttle vector (Fig. 2B) suggest that the copy number of the shuttle vector, and thus that of *dcas9*, did not vary among these strains. Therefore, the mutations in the promoter and RBS decreased basal *dcas9* expression levels as intended.

Next, we determined appropriate induction conditions using strain CJW_Bb242 given it displayed the highest basal expression of *dcas9* (Fig. 2A). In this strain, induction of *dcas9* expression with 1 mM IPTG, a concentration known to elicit maximal expression from P*_pQE30_* in B. burgdorferi (32), resulted in slower growth in liquid culture and in semisolid Barbour-Stoenner-Kelly (BSK)-agarose plates compared to the no-IPTG condition (data not shown). This growth defect is not observed during the induction of expression of other genes (32). It is therefore likely due to overproduction of dCas9 and subsequent toxic effects associated with excessive nonspecific DNA binding, as observed in other bacteria (28, 40). Importantly, lowering the concentration of IPTG to 0.1 mM resulted in no discernible growth defect (Fig. 2C). We therefore used 0.1 mM IPTG to induce *dcas9* expression in all subsequent experiments.

We then measured the magnitude of induction of *dcas9* expression by IPTG. The highest magnitude, ∼400-fold, was observed in strain CJW_Bb430, which carries a chromosomal copy of the *dcas9-lacI* cassette (Fig. 2D). Among the shuttle vector-encoded *dcas9* versions, we observed the highest induction of ∼40-fold in the case of the unmutated promoter (Fig. 2D, strain CJW_Bb242). The RBSmut version displayed an ∼15-fold induction, while the −10 promoter mutation allowed only a 2-fold induction (Fig. 2D). The genetically linked *lacI* gene experienced little change in its expression level in response to IPTG induction (Fig. 2E), as expected.

Finally, we imaged the strains after 2 days of exposure to IPTG and saw no notable changes in cell morphology between induced and uninduced cultures (Fig. 2F), further supporting the notion that these levels of *dcas9* induction are not toxic to the cells.

### B. burgdorferi genes targeted by CRISPRi.

To test our CRISPRi platform, we targeted the following four B. burgdorferi genes: *flaB*, *ftsI*, *rodA*, and *mreB*. Depletion of their protein products is expected to yield morphological phenotypes, which are easily observable by microscopy. The *flaB* gene, which encodes flagellin, the major structural component of periplasmic flagella, is required for motility and for generating the characteristic flat wave morphology of B. burgdorferi cells (41, 42). FtsI is a transpeptidase that is required for septal peptidoglycan synthesis during cell division; its inhibition causes cell filamentation in E. coli (43–46). RodA, encoded by the *rodA* (*mrdB*) gene, is a transglycosylase active during lateral peptidoglycan synthesis in many rod-shaped bacteria (47). This lateral wall elongation is orchestrated by MreB, a bacterial actin homolog (48). When rod-shaped bacteria such as E. coli or C. crescentus lose RodA function, they grow into rounder shapes (49–51), as they do following MreB inactivation or depletion (52, 53).

Across them, the *flaB*, *rodA*, *mreB* and *ftsI* genes span 2 orders of magnitude of native expression levels (54), a range that covers a large subset of B. burgdorferi genes expressed during exponential growth *in vitro* (see Fig. S2A in the supplemental material). Furthermore, these genes are either the last gene in an operon or encode a monocistronic mRNA (Fig. S2B), rendering polar effects of CRISPRi unlikely. We designed sgRNAs targeting these four genes and cloned them either in sgRNA shuttle vectors or in all-in-one CRISPRi shuttle vectors (Table 1). These sgRNAs recognize sequences found in either the 5′ UTR or the protein-coding region of the targeted gene, near the translational start site (Fig. S2B). We introduced the sgRNA-expressing shuttle vectors into appropriate B. burgdorferi host strains, generating CRISPRi strains for depletion of FlaB, FtsI, MreB, and RodA (Table 2).

### Basal and induced CRISPRi activity in B. burgdorferi.

In the absence of IPTG, clone generation, growth, and cell morphology were normal for the CRISPRi strains targeting *flaB*, regardless of the version of the CRISPRi platform used (see Table S2 in the supplemental material). In contrast, upon transforming B. burgdorferi B31-A3-68-Δ*bbe02*::*P_flgB_-aphI* (also known as “K2”) (33) (Table 2) with all-in-one CRISPRi shuttle vectors containing an unmutated P*_pQE30_* promoter and targeting *rodA*, *mreB*, or *ftsI*, we observed phenotypes consistent with significant basal CRISPRi activity (Table S2). For example, we were unable to generate clones when transforming B. burgdorferi K2 with shuttle vectors targeting *mreB* (Table S2). We also observed delays in appearance of colonies in BSK-agarose plates when using shuttle vectors targeting *rodA* (Table S2). Even in the absence of IPTG, cells of the RodA depletion strain (CJW_Bb346) sometimes had normal flat wave morphologies but often displayed bulging (see Fig. S3 in the supplemental material), consistent with a RodA depletion phenotype. We believe that the high basal plasmid-based expression of *dcas9* from the unmutated P*_pQE30_* promoter (Fig. 2A), combined with the constitutive expression of the sgRNA, led to formation of enough dCas9-sgRNA complexes to repress transcription of the targeted genes even in the absence of IPTG. When targeting *rodA*, introduction of the −10AC1, −10AC2, or −10AC12 mutations (Fig. S1F) into the P*_pQE30_* promoter in the context of the all-in-one CRISPRi shuttle vector did not fully abrogate the CRISPRi phenotype in the absence of IPTG induction (Table S2). We did not further analyze the strains carrying these constructs. In contrast, the −10TC or RBSmut versions of the all-in-one CRISPRi shuttle vector (Fig. 1H and Table 2), as well as the chromosomal *dcas9* version of the CRISPRi platform, allowed generation of strains that displayed no or weak phenotypic evidence of basal CRISPRi activity (Table S2).

**FIG 3:**
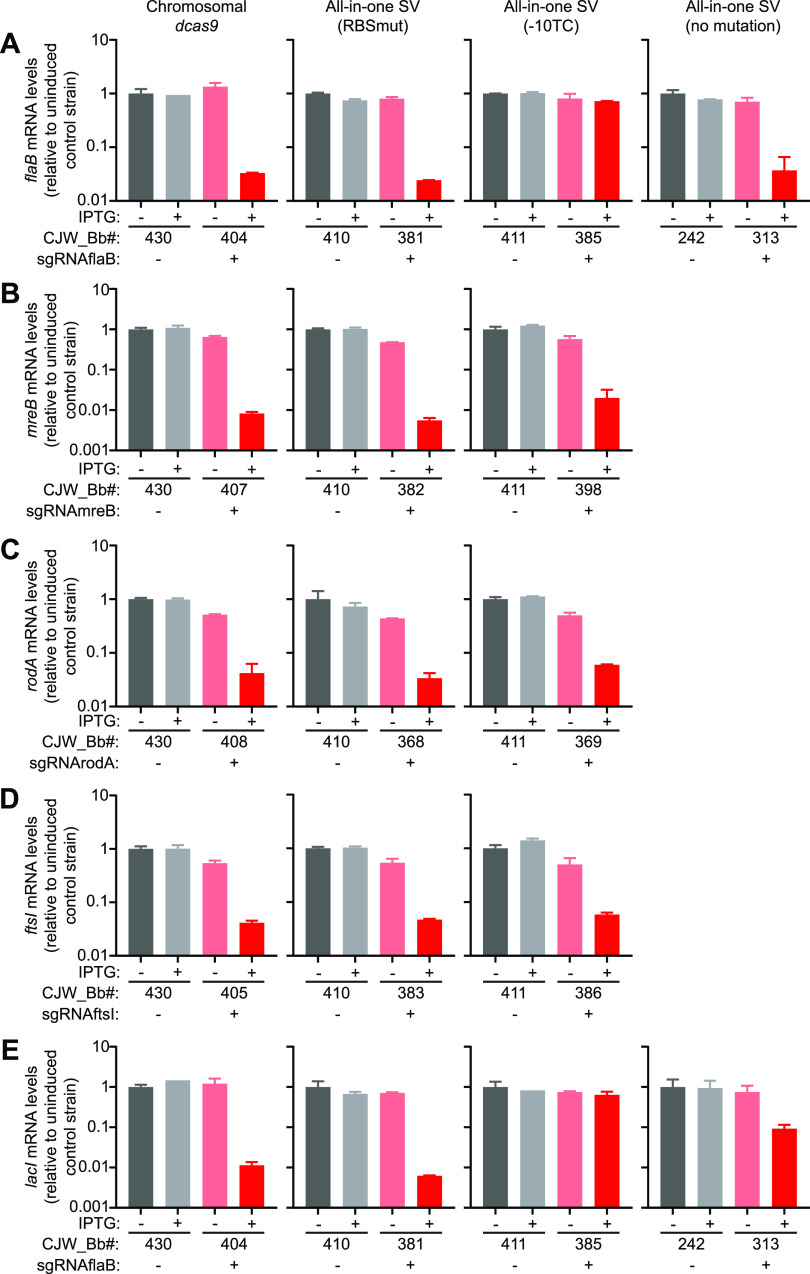
Effect of CRISPR interference (CRISPRi) on targeted gene mRNA levels. (A) *flaB*, (B) *mreB*, (C) *rodA*, (D) *ftsI*, and (E) *lacI* mRNA levels measured in the indicated control strains (gray) and CRISPRi depletion strains (pink and red) after 1 day of growth with or without IPTG. Shown are the means ± standard deviations measured from two cultures. The version of the CRISPRi platform carried by each set of strains is indicated above the corresponding column of graphs. SV, shuttle vector.

We next characterized CRISPRi efficiency by qRT-PCR. In the absence of an sgRNA, the mRNA levels of *flaB*, *ftsI*, *mreB*, or *rodA* were not affected by IPTG addition (Fig. 3 and Fig. S4 in the supplemental material, gray bars). In the absence of IPTG, *flaB* mRNA levels were only slightly, if at all, reduced in strains constitutively expressing an sgRNA targeting the *flaB* gene (sgRNAflaB) compared to those in the corresponding control strains lacking sgRNAflaB (Fig. 3A and Fig. S4A). In contrast, sgRNAs binding to *ftsI*, *mreB*, or *rodA* decreased their targets’ mRNA levels by ∼40% to ∼60% of those in the control strains, even without IPTG induction of *dcas9* expression (Fig. 3B to D and Fig. S4B to D). These lower mRNA levels appeared to be relatively well tolerated by the cells, as fewer than ∼1% of the cells in each population displayed morphological defects based on visual inspection (Table S2).

IPTG induction of *dcas9* expression for 24 h in three of the four strains carrying sgRNAflaB (CJW_Bb404, CJW_Bb381, and CJW_Bb313) depleted *flaB* mRNAs by at least 95% of the levels measured in the corresponding control strains in the absence of IPTG (Fig. 3A). The depletion was maintained over 2 days of IPTG exposure (Fig. S4A). Induction of *dcas9* expression in these strains also depleted *lacI* mRNAs (Fig. 3E and Fig. S4E). This was expected, as *lacI* expression is controlled by P*_flaB_*, which contains the 5′ UTR of the *flaB* gene (7) and therefore the CRISPR site targeted by our sgRNA. In the remaining strain carrying sgRNAflaB (CJW_Bb385), *flaB* and *lacI* mRNA levels decreased noticeably less after IPTG induction (Fig. 3A and 3E and Fig. S4A and S4E). Presumably, the weak induction of *dcas9* expression from the mutated (−10TC) *P_pQE30_* promoter (Fig. 2D) is insufficient to cause repression of both the *flaB* and *lacI* genes, located on the chromosome and multicopy plasmid, respectively.

We also quantified *ftsI*, *mreB*, or *rodA* mRNA levels in the corresponding depletion strains grown in the presence of IPTG and compared them to controls lacking an sgRNA (Fig. 3B to D and Fig. S4B to D). In all strains, regardless of the version of the CRISPRi system present, induction of *dcas9* expression considerably depleted the mRNA of the targeted genes. The magnitude of the depletion after 1 day of IPTG exposure ranged from ∼95% for *rodA* and *ftsI* to ∼99% for *mreB* (Fig. 3B to D). Such low mRNA levels were still observed after 2 days following IPTG addition to the cultures (Fig. S4B to D).

### Phenotypic characterization of CRISPRi-mediated flagellin depletion.

**FIG 4:**
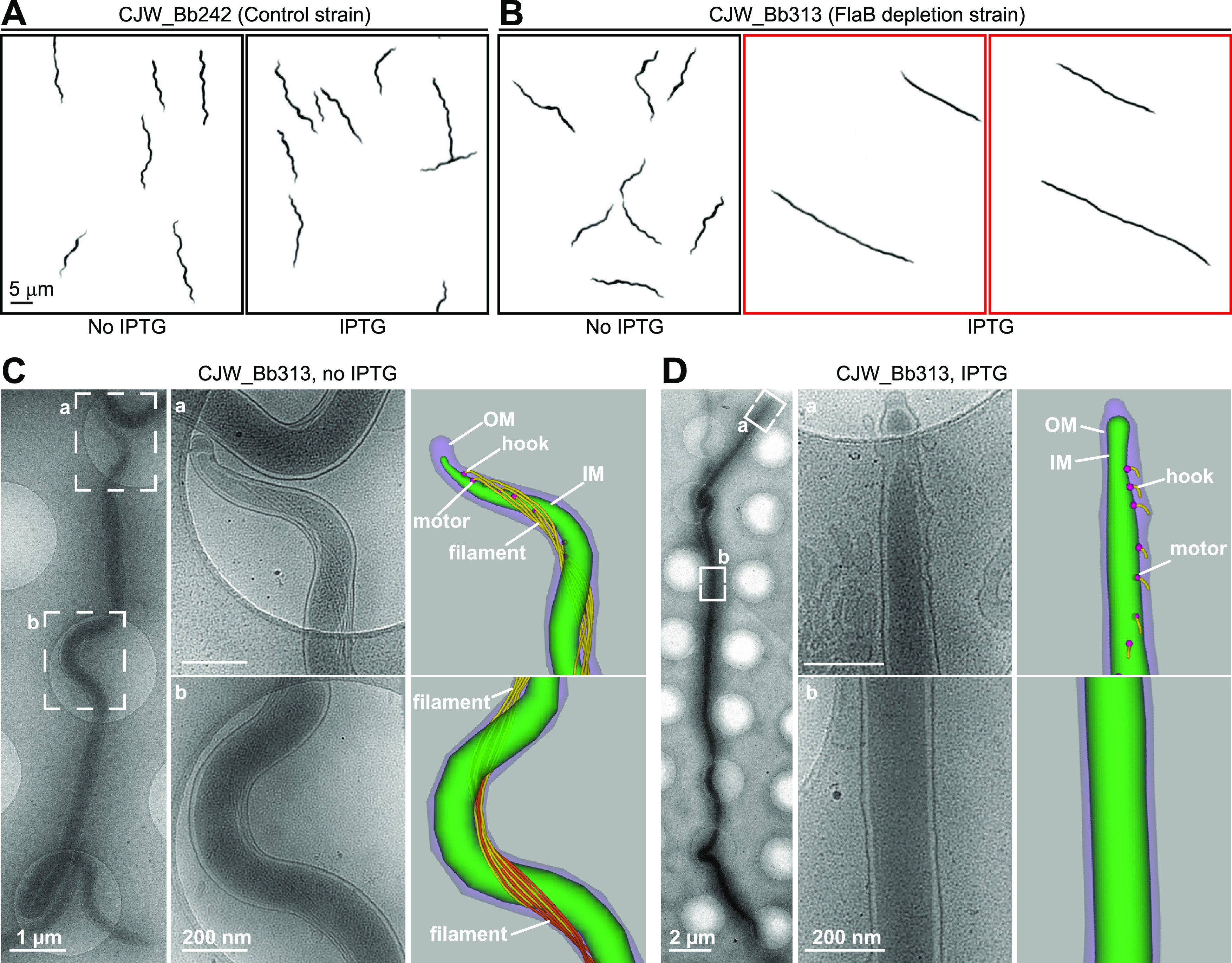
Phenotypic characterization of flagellin depletion. (A and B) Inverted darkfield images of strain CJW_Bb242 (A) or CJW_Bb313 (B) grown in the absence or presence of IPTG for 2 days. The flagellin depletion phenotype is highlighted by a red outline. (C) Cryo-electron tomography (cryo-ET)-based detection of periplasmic flagella in a cell of strain CJW_Bb313 grown in the absence of IPTG. (Left) Low-magnification view of the entire cell. (Center) High-magnification views of the tip (a) and center (b) of the cell. (Right) Three-dimensional segmentation of the tip (panel a) and center (panel b) regions of the cell. In panel b, the flagella from one end of the cell are shown in yellow and the flagella from the other end are shown in orange; see also Fig. S6A in the supplemental material. (D) Flagellin depletion assessed by cryo-ET in a cell of strain CJW_Bb313 after 2 days of IPTG exposure. (Left) Low-magnification view of the entire cell. (Center) High-magnification views of the tip (a) and center (b) of the cell. (Right) Three-dimensional segmentation of the tip (panel a) and center (panel b) of the cell.

Despite the very high level of expression of B. burgdorferi flagellin (Fig. S2A), CRISPRi-mediated depletion of this protein was able to elicit a partial loss-of-function phenotype. Flagella impart to B. burgdorferi cells their motility and flat wave morphology (41, 42), which we readily observed in uninduced or induced cells of control strains carrying no sgRNA (Fig. 4A and Movies S1 and S2 and Fig. S5A to C in the supplemental material), or in uninduced cells of strains carrying sgRNAflaB (Fig. 4B and Movie S3 and Fig. S5A to D in the supplemental material). After 2 days of IPTG exposure, cells of strain CJW_Bb313, which express sgRNAflaB from P*_pQE30_* on the shuttle vector, displayed various degrees of motility defects (see Movies S4 to S8 in the supplemental material). While most cells retained some flat wave morphology and twitching ability, they appeared straightened compared to their control counterparts (Fig. 4B). For the cells that were still able to move, most often the movement was slowed (Movie S4), reduced to a twitching pattern (Movies S5 and S6), or localized at the cell pole region (Movie S6). Some cells displayed little to no evidence of flagellum-based motion and instead appeared to simply display Brownian motion or drift in the medium (Movies S7 and S8). Some of these cells were almost completely straight, except for the occasional kink at the division site (Movie S8).

Expressing sgRNAflaB from seven different promoters of varied strengths resulted in a similar overall straightening of the cell body (Fig. 4B and Fig. S1D and S5D and Table 2 and Table S1). Our results therefore suggest that our CRISPRi system is robust to variation in sgRNA levels in B. burgdorferi. We also observed an overall straightening of the cell in IPTG-induced strains that carry either the chromosomal *dcas9* (CJW_Bb404; Fig. S5A) or the all-in-one CRISPRi shuttle vector with the RBS P*_pQE30_* mutation (CJW_Bb381; Fig. S5B). This is consistent with the significant *flaB* mRNA depletion observed in these strains (Fig. 3A and Fig. S4A). As expected, cells from strain CJW_Bb385, in which *flaB* transcript levels remained largely unaffected by IPTG induction (Fig. 3A and Fig. S4A), displayed normal morphology (Fig. S5C).

Flagellin depletion is expected to occur gradually over generations following IPTG induction, which could explain the mixed phenotypes we observed. In B. burgdorferi, multiple flagella are anchored near each cell pole (55, 56). They form bundles that extend from their subpolar anchors toward the center of the cell, where they overlap. We reasoned that retention of some wave-like cell shape in otherwise poorly motile or nonmotile, flagellin-depleted cells may be caused by a decrease in the length or number of flagella. In this scenario, fewer flagella might exert enough force to bend the cell into a reduced-amplitude wave-like shape but not enough force to generate translational motion. To test these potential explanations, we imaged frozen-hydrated B. burgdorferi cells by cryo-electron tomography (cryo-ET). In the absence of IPTG, the cellular ultrastructure was indistinguishable from that previously reported (56, 57). Multiple flagella were attached near the cell poles (Fig. 4C, panel a, and Fig. S6A in the supplemental material) and the flagellar bundles extended toward the middle of the cell, where they overlapped (Fig. 4C, panel b). In contrast, after exposure to IPTG for 2 days, the cells had no detectable flagellar filament around midcell (Fig. 4D and Fig. S6B, panel b), explaining the observed motility defects. At pole-proximal regions, flagellar hooks could be readily detected (Fig. 4D and Fig. S6A, panel a). However, no flagellar filament (Fig. 4D) or shorter filaments (Fig. S6A) could be detected at these subcellular regions, consistent with a strong depletion of FlaB.

### FtsI involvement in B. burgdorferi cell division.

**FIG 5:**
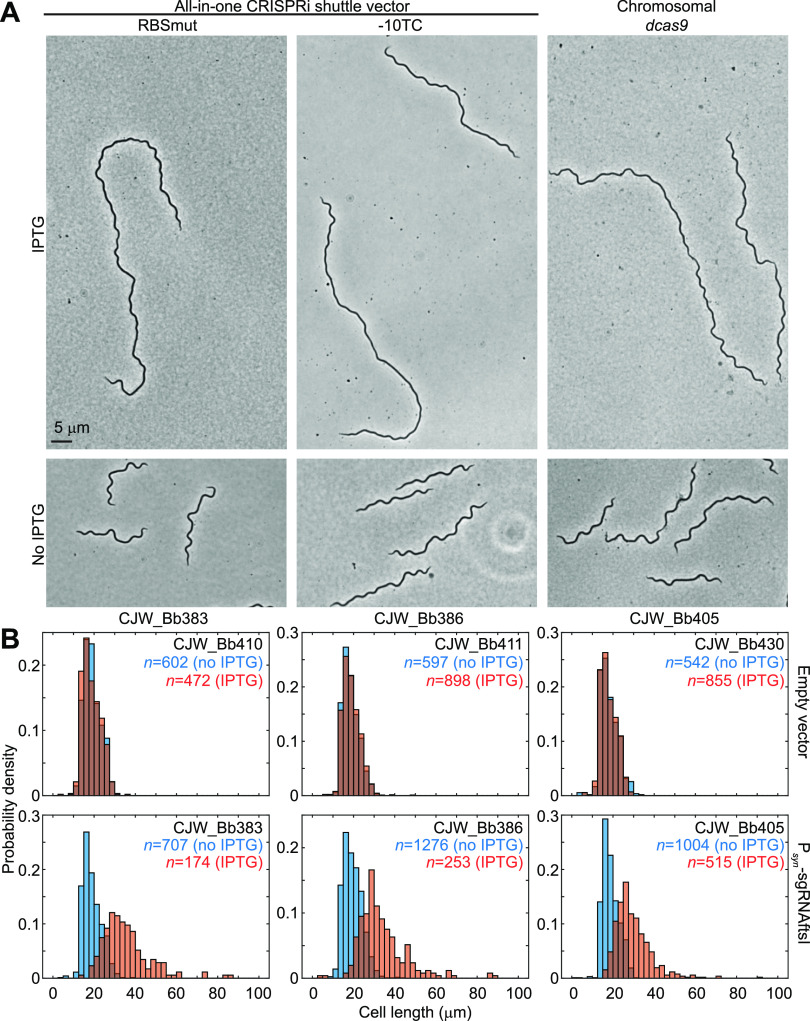
Phenotypic characterization of FtsI depletion. (A) Phase-contrast micrographs of cells from cultures of strains expressing sgRNAftsI after 2 days of *dcas9* induction with IPTG or in the absence of IPTG. (B) Histograms depicting distributions of cell lengths measured in induced (0.1 mM IPTG for 2 days, orange) or uninduced (no IPTG, blue) cultures of the noted strains. The strains expressed either no sgRNA (top row) or sgRNAftsI (bottom row).

Knockdown of *ftsI* expression by CRISPRi for 2 days elicited significant cell filamentation in all the strains tested, in contrast to the uninduced cells of the same strains (Fig. 5A). Cell filamentation required expression of sgRNAftsI, as shown by cell length quantification (Fig. 5B). Cells almost 100 μm long, which is about five times the average length of a B. burgdorferi cell, were detected (Fig. 5B, bottom). We note that when immobilized between an agarose pad and a coverslip, as in our microscopy setup, longer cells are more likely to cross themselves or other cells, bend at a tight angle, or lie partly outside the field of imaging. In such cases, automated cell outline generation with the Oufti software package (58) was not possible, and such cells were excluded from our measurements. Therefore, the cell length distributions measured in FtsI-depleted cultures likely underestimate the extent of cell filamentation present in the population. Regardless, our results implicate FtsI in B. burgdorferi cell division.

### Rod morphogenesis functions of MreB and RodA in B. burgdorferi.

**FIG 6:**
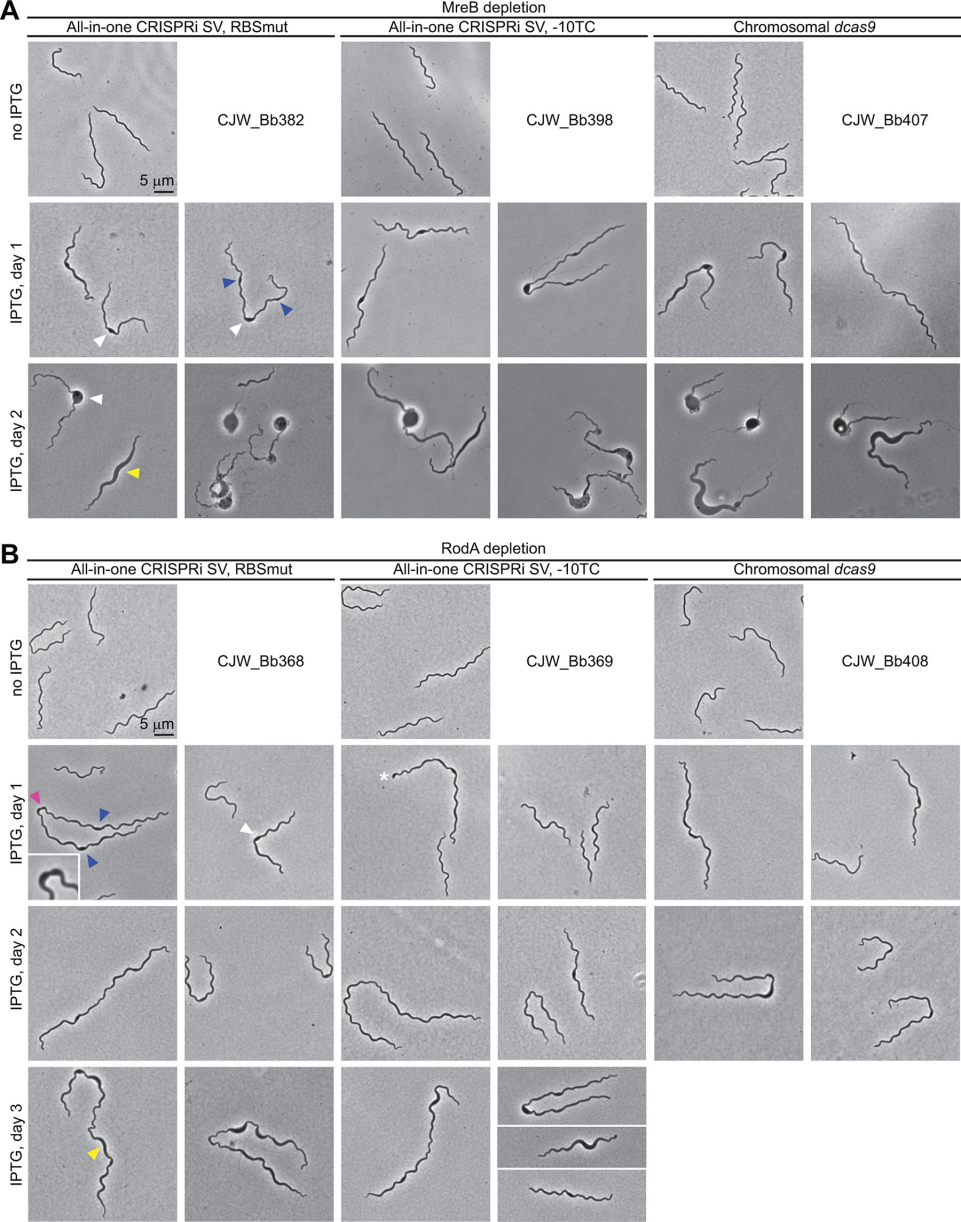
Phenotypic characterization of MreB and RodA depletion. (A and B) Phase contrast micrographs of cells from cultures of CRISPRi strains targeting *mreB* (A) or *rodA* (B). The marks show the following phenotypes: white arrowheads, cell bulging localized at midcell; blue arrowheads, cell bulging localized at approximately the 1/4 and 3/4 positions along the cell length; yellow arrowheads, cell widening extending along the cell length; pink arrowhead, a widened division site, shown in greater detail in the inset; and white asterisk, cell displaying an enlarged pole. SV, shuttle vector.

We imaged the MreB and RodA depletion strains before IPTG addition and daily after induction. We found that 1 day of MreB depletion was enough for significant cell bulging to develop (Fig. 6A). Bulging occurred predominantly at midcell (Fig. 6A, white arrowheads). In some cells, especially long ones, bulging was also apparent at ∼1/4 and ∼3/4 locations along the length of the cell (Fig. 6A, blue arrowheads). Our laboratory has previously shown that new peptidoglycan synthesis occurs at these subcellular locations in members of the *Borrelia* genus (59). The bulging phenotype at these locations therefore suggests that MreB is important for maintaining a constant cell width during insertion of peptidoglycan material at these specific sites (48). Furthermore, less pronounced cell body widening outside these discrete locations, but encompassing longer segments of the cells, was also observed (Fig. 6A, yellow arrowheads). After 2 days of MreB depletion (Fig. 6A), the bulging phenotype became further exacerbated, with larger bulges at midcell and more pronounced laterally spread cell body widening compared to the 1-day time point. Overall, our findings establish a key role for MreB in B. burgdorferi cell morphogenesis.

RodA depletion also elicited cell widening in B. burgdorferi (Fig. 6B). In the absence of IPTG, cells looked normal (Fig. 6B). Upon IPTG induction, bulging could be observed at midcell (Fig. 6B, white arrowhead) and, occasionally, at the 1/4 and 3/4 locations (Fig. 6B, blue arrowheads). Midcell bulging nevertheless permitted cell division to occur in some instances, as evidenced by the occurrence of deep constriction within a bulge (Fig. 6B, pink arrowhead and inset) and the occasional presence of rounded poles (Fig. 6B, white asterisk).

## DISCUSSION

In this study, we adapted the CRISPRi system for use in B. burgdorferi and tested its capabilities by targeting the motility and/or cell morphogenesis genes *flaB*, *ftsI*, *mreB*, and *rodA*. These genes span a broad range of native expression levels (see Fig. S2A in the supplemental material) (54), suggesting that CRISPRi will have broad applicability in the study of B. burgdorferi.

We created several variations of the CRISPRi platform. The all-in-one CRISPRi shuttle vectors can be introduced into any transformable B. burgdorferi strain, allowing comparison of behaviors across multiple genetic backgrounds and facilitating the pairing of CRISPRi-based gene depletion with other genetic methods. For example, using our system, the effects of depleting one gene product can be easily compared among otherwise isogenic strains that are wild-type, mutated, or complemented at a second gene locus. We further facilitated such pairing by generating CRISPRi shuttle vectors carrying each of five compatible antibiotic resistance markers (Table 1).

Another variant of our CRISPRi platform relies on two elements, a *dcas9-lacI* cassette stably integrated into the chromosome of a transformable derivative of the type strain B31 and an sgRNA expressed from a shuttle vector. The copy number of chromosomally expressed *dcas9* is expected to covary with the copy number of the targeted locus, whether it is located on the chromosome or on an endogenous plasmid. Prior studies have documented an ∼1:1 copy number ratio between various endogenous plasmids and the chromosome (34, 35, 60).

A common characteristic of all versions of our CRISPRi platform is that they only require a single shuttle vector transformation step. B. burgdorferi B31-derived strains that lack restriction modification enzymes are transformed by shuttle vectors at a higher frequency, in the range of 10^−4^ to 10^−5^ (33), than the ∼10^−7^ frequency obtained using suicide vectors (7, 9). Thus, our CRISPRi platform offers an efficient complementary tool to homologous recombination-based methods for interrogation of gene function in B. burgdorferi.

In characterizing the CRISPRi platform, we found that low basal *dcas9* expression in the absence of IPTG, combined with sizeable induction of *dcas9* expression upon IPTG addition, appears to be important for broad functionality of the approach. Such is the case for the RBSmut version of the all-in-one CRISPRi shuttle vector and for the chromosomally encoded *dcas9.* These CRISPRi platform versions yielded depletion phenotypes for all the genes we targeted. We recommend these versions in future CRISPRi experiments aimed at downregulating the expression of other genes.

Our CRISPRi approach allowed us to provide genetic insight into cell morphogenesis in B. burgdorferi. To our knowledge, neither deletion mutants nor transposon insertion mutants have been reported for B. burgdorferi *ftsI*, *mreB*, or *rodA* (61), potentially because these genes are essential for viability. Our inability to obtain clones while targeting *mreB* using the CRISPRi platform version that had the highest basal expression of *dcas9* supports this notion for *mreB*. We also tried to use A22 and MP265, two known small-molecule inhibitors of MreB (62–66), to study the function of this cytoskeletal protein in B. burgdorferi. Our attempts, however, proved unsuccessful, as B. burgdorferi appears to be resistant to chemical inhibition of MreB (see supplemental text and Fig. S7 in the supplemental material). Other examples of inhibitors widely used in other bacteria but not effective in B. burgdorferi include the transcription inhibitor rifampin, which acts on RpoB (67), and the peptidoglycan precursor synthesis inhibitor fosfomycin, which acts on MurA (43, 68). This list of important cellular functions that are apparently refractive to chemical inhibition in B. burgdorferi further underscores the utility of our easy-to-use and rapid CRISPRi genetic approach.

When we depleted FtsI, cells elongated into filaments, consistent with a conserved involvement of this protein in cell division (45, 69, 70). Depletion of RodA and MreB led to cell widening, reminiscent of loss-of-function phenotypes observed in other bacteria (50, 51, 53, 62, 70, 71). These results fit with the current model in which MreB orients lateral cell wall synthesis in such a way that a constant cell width is generated and propagated during growth (48). RodA, in turn, is an elongation-specific transglycosylase (47) that contributes to the wall biosynthetic function organized by MreB (43, 48). Thus, the cell widening and bulging associated with MreB and RodA depletion in B. burgdorferi indicates that rod morphogenesis is controlled in this spirochete, at least in part, by some of the same actors as in other rod-shaped bacteria.

Bulging secondary to MreB depletion overwhelmingly occurred at sites of new peptidoglycan synthesis (59), primarily the midcell, but also the 1/4 and 3/4 positions along the length of the cells. The pattern of new peptidoglycan insertion in *Borrelia* species is peculiar compared to that seen in other bacteria, including other spirochetes such as those belonging to the *Treponema* and *Leptospira* genera (59). Those spirochetes elongate by inserting new peptidoglycan along the entire length of their cells. We do not yet know what mechanisms control the *Borrelia* pattern of new wall insertion. However, our observation that morphologic defects secondary to MreB depletion matched the sites of new peptidoglycan insertion suggests a connection between MreB function and B. burgdorferi’s unusual pattern of cell wall growth.

Our results highlight the usefulness of a CRISPRi genetic approach. While this approach does not replace traditional homologous recombination-based methods, it offers some advantages as a complementary approach due to its procedural ease, speed, efficiency, and scalability. CRISPRi-based methods have proven to be particularly useful for the phenotypic characterization of genes essential for viability, for the simultaneous repression of multiple genes, and for high-throughput genome-wide studies (18, 19, 24, 28).

## MATERIALS AND METHODS

### Bacterial growth conditions.

All E. coli strains were grown on LB agar plates or in Super Broth (35 g/liter bacto-tryptone, 20 g/liter yeast extract, 5 g/liter NaCl, and 6 mM NaOH) liquid cultures at 30°C, with shaking. The following final concentrations of antibiotics were used for E. coli growth and selection: kanamycin at 50 μg/ml, gentamicin at 15 to 20 μg/ml, spectinomycin or streptomycin at 50 μg/ml, rifampin at 25 μg/ml for liquid selection or 50 μg/ml for growth on plates, and hygromycin B at 100 to 200 μg/ml. After heat shock or electroporation, E. coli transformants were allowed to recover in SOC medium (20 g/liter tryptone, 5 g/liter yeast extract, 10 mM NaCl, 2.5 mM KCl, 10 mM MgCl_2_, 10 mM MgSO_4_, and 20 mM glucose) for 1 h with shaking at 30°C, before plating. E. coli strain MC1000 (72) was grown overnight in LB liquid culture at 37°C with shaking, then diluted 1:1,000 in BSK-II medium (see below), grown for another 3 h, and finally treated with 50 μM MP265 or A22 (63) from a 1,000× dimethyl sulfoxide (DMSO) stock, or with DMSO alone, for 1 h.

B. burgdorferi strains are listed in Table 2. They were grown at 34°C in a humidified incubator under 5% CO_2_ atmosphere (11, 13, 14). Liquid cultures were grown in Barbour-Stoenner-Kelly (BSK)-II medium (11), containing 50 g/liter bovine serum albumin (catalog no. 810036; Millipore), 9.7 g/liter CMRL-1066 (catalog no. C5900-01; US Biological), 5 g/liter Neopeptone (catalog no. 211681; Difco), 2 g/liter Yeastolate (catalog no. 255772; Difco), 6 g/liter HEPES (catalog no. 391338; Millipore), 5 g/liter glucose (catalog no. G7021; Sigma-Aldrich), 2.2 g/liter sodium bicarbonate (catalog no. S5761; Sigma-Aldrich), 0.8 g/liter sodium pyruvate (catalog no. P5280; Sigma-Aldrich), 0.7 g/liter sodium citrate (catalog no. BP327; Fisher Scientific), 0.4 g/liter *N*-acetylglucosamine (catalog no. A3286; Sigma-Aldrich) (pH 7.60), and 60 ml/liter heat-inactivated rabbit serum (catalog no. 16120; Gibco). BSK-1.5 medium contained 69.4 g/liter bovine serum albumin, 12.7 g/liter CMRL-1066, 6.9 g/liter Neopeptone, 3.5 g/liter Yeastolate, 8.3 g/liter HEPES, 6.9 g/liter glucose, 6.4 g/liter sodium bicarbonate, 1.1 g/liter sodium pyruvate, 1.0 g/liter sodium citrate, 0.6 g/liter *N*-acetylglucosamine (pH 7.50), and 40 ml/liter heat-inactivated rabbit serum. Antibiotics were used at the following final concentrations for both liquid cultures and plates: kanamycin at 200 μg/ml, gentamicin at 40 μg/ml, streptomycin at 100 μg/ml, and hygromycin B at 300 μg/ml, respectively (6–8, 73). IPTG (catalog no. AB00841; American Bioanalytical) was used at 0.1 mM final concentration from a stock of 1 M in water. Unless otherwise specified, cultures were always maintained in exponential growth, with culture densities kept below ∼5 × 10^7^ cells/ml. Cell density was determined by direct counting under darkfield illumination of samples placed in disposable hemocytometers, as previously described (6).

### B. burgdorferi transformation and clone isolation.

Competent cells were generated as previously described (9). B. burgdorferi cultures containing exponentially growing cells were allowed to reach densities between 2 × 10^7^ cells/ml and ∼1 × 10^8^ cells/ml. Next, the cultures were centrifuged for 10 min at 10,000 × *g* and 4°C in 50-ml conical tubes, and the medium was removed by aspiration without disturbing the cell pellets. Cell pellets from 2 or 3 tubes were combined by resuspension in 40 ml cold electroporation solution (EPS) containing 93.1 g/liter sucrose (catalog no. AB01900; American Bioanalytical) and 150 ml/liter glycerol (catalog no. AB00751; American Bioanalytical) and centrifuged again. A second wash in 1 ml cold EPS was then performed, after which the cells were pelleted and resuspended in 50 to 100 μl of cold EPS for each 100 ml of initial culture. The competent cells were placed on ice or frozen at −80°C until use.

For cell transformation with a shuttle vector (4, 74), 25 to 50 μg of plasmid (in water) were mixed with 50 to 100 μl of competent cells of strain B31-A3-68-Δ*bbe02*::P*_flgB_*-*aphI* “K2” (33), B31 e2 (75), or CJW_Bb362, and electroporated (2.5 kV, 25 μF, 200 Ω, and 2 mm gap cuvette). Electroporated cells were immediately recovered in 6 ml BSK-II and incubated overnight at 34°C. To insert the *dcas9-lacI* cassette into the chromosome, the pKIKan_idCas9_Chr_center plasmid (75 μg) was linearized in a 500-μl reaction volume containing 100 units of ApaLI enzyme for 4 to 6 h at 37°C in CutSmart buffer (New England Biolabs). The DNA was then ethanol precipitated as previously described (76). The DNA pellet was dried in a biosafety cabinet, then resuspended in 25 μl water, chilled on ice, mixed with 100 μl of competent cells of the B31-A3-68-Δ*bbe02*::P*_flaB_-aadA* “S9” strain (33), and electroporated. The cells were allowed to recover overnight in 6 ml of BSK-II medium.

After overnight recovery, electroporated B. burgdorferi cells were plated in semisolid BSK-agarose medium. Three volumes of BSK-1.5 medium containing appropriate concentrations of antibiotics were equilibrated at 34 to 37°C, then briefly brought up to 55°C in a water bath and mixed with two volumes of 1.7% (wt/vol) agarose solution in water, also preequilibrated at 55°C. The mixed BSK-antibiotics-agarose solution (25 ml/plate) was added to 10-cm petri dishes containing aliquots of the electroporated B. burgdorferi cells and gently swirled to mix. The plates were chilled to room temperature for ∼30 min in a biosafety cabinet, then transferred to a humidified 5% CO_2_ incubator kept at 34°C for 1 to 3 weeks until colonies became clearly visible by eye. Alternatively, selection was performed in liquid culture by mixing 1 ml of electroporated cells with 5 ml of BSK-II medium containing appropriate concentrations of selective antibiotics. Beginning on the fifth day of selection, the cultures were visually inspected for growth by darkfield microscopy. Clones were isolated from these nonclonal, selected populations by limiting dilution in 96-well plates, as previously described (6) or by semisolid BSK-agarose plating, as described above. Agarose plugs containing single colonies were removed from the plates and expanded in 6 ml of BSK-II medium containing selective antibiotics. Insertion of the *dcas9-lacI* cassette at the desired chromosomal location in strain CJW_Bb362 was confirmed by PCR analysis of isolated total genomic DNA (DNeasy blood and tissue kit, Qiagen) using the following primer pairs: NT424 and NT425 (amplifies across the insertion site), NT591 and NT592 (amplifies within the kanamycin resistance gene *aphI*), NT681 and NT682 (amplifies within *lacI*), and NT683 and NT684 (amplifies within *dcas9*) (see Table 3 for primer sequences). Except for strain CJW_Bb122 (which is not derived from an infectious strain), the plasmid complement of each isolated clone was determined using primer pairs previously described (77) and DreamTaq Green DNA polymerase (Thermo Fisher Scientific). Each clone was confirmed to contain all B31-specific plasmids, except lp5, cp9, and lp56, which are also absent from the parental strains (33). The clones were invariantly maintained in exponential growth and frozen at passage one or two.

**TABLE 3:** Synthetic DNA sequences used for cloning

Name	Sequence (5′ to 3′)^*a*^
Gene block sequence	
MS0	AGCTATGACCATGATTACGAATTCGAGCTCGAATTCTAAAGATCTTTGACAGCTAGCTCAGTCCTAGGTATAATACTAGTGGAAGAGCGAGCTCTTCCGTTTTAGAGCTAGAAATAGCAAGTTAAAATAAGGCTAGTCCGTTATCAACTTGAAAAAGTGGCACCGAGTCGGTGCTTTTTTTGAAGCTTGGGCCCGAACAAAAACTCAAAGCTTGGC ACTGGCCGTCGTTTTACAAC
Primers used for cloning	
MS1	TTTTGAAGCTTGGGCCCGAACAAAAACTCAAAGCTTGGCACTGGCCGTCGTTTTACAAC
MS2	TGAGCTAGCTGTCAAAGATCTTTAGAATTCGAGCTCGAATTCGTAATCATGGTCATAGCT
MS3	CAGAATTGGAAAGTATTTTATTGCAACACCCAGCTCTTCCGTTTTAGAGCTAGAAATAGC
MS4	GATCATATTTGTCTATAAGTGTTGACTTTGGCTCTTCCACTAGTATTATACCTAGGAC
MS5	GTCCTAGGTATAATACTAGTGGAAGAGCCAAAGTCAACACTTATAGACAAATATGATC
MS6	GCTATTTCTAGCTCTAAAACGGAAGAGCTGGGTGTTGCAATAAAATACTTTCCAATTCTG
MS7	AGTGAAAATTTAAATTTCTGACTT
MS8	AACAAGTCAGAAATTTAAATTTTC
MS9	AGTGATATCTATTGCAACAACAGA
MS11	ATAGCTAAGCCTATTGAGTATTTCTTATCCATATGTAATTTCTCCTCTTTAATGAATTCT
MS12	ATTGATTTGAGTCAGCTAGGAGGTGACTAATAAAAGCTTGATCAGATCTCAGCTTTTT
MS13	AGAATTCATTAAAGAGGAGAAATTACATATGGATAAGAAATACTCAATAGGCTTAGCTAT
MS14	AAAAAGCTGAGATCTGATCAAGCTTTTATTAGTCACCTCCTAGCTGACTCAAATCAAT
MS15	GACAGGATGAGGATCGTTTCGCATGATTGCGCCTTCTTGACGAGTTCTTCTGAATTG
MS16	CAATTCAGAAGAACTCGTCAAGAAGGCGCAATCATGCGAAACGATCCTCATCCTGTC
NT89	CTCGGTCTATTCTTTTGATTTACATGACCAAAATCCCTTAACG
NT90	CGTTAAGGGATTTTGGTCATGTAAATCAAAAGAATAGACCGAG
NT170	TATCGGCCGCATGGCTTGTTATGACTG
NT267	TATGAGCTCCTAAGTAATGCAAATAATAATCCCACATTG
NT268	GACACTAGTCACTATTTTAAATTCCAGGCGATAAAACC
NT269	CACCTGCAGCTAGTTTAAAATTTATTTATCTTGATTATTATTTTTTATGGAG
NT270	TATCTCGAGCGAATCAAGTATCAACTTCAATTCTTGAG
NT350	CTAGTAACCCGGGTAAAAAAACAAAAGATCCTTTAAAGGATCTTTTGTTAATA
NT351	CCGGTATTAACAAAAGATCCTTTAAAGGATCTTTTGTTTTTTTACCCGGGTTA
NT352	ACTTAAGGGACCTGCAAGTAGTGCAATTTGTTGATGGCCTA
NT353	TAGGCCATCAACAAATTGCACTACTTGCAGGTCCCTTAAGT
NT354	CCACGGCCGGAATTCTAAAGATCTTTGACAGCTAGCTCAGTCC
NT355	TATGGCGCGCCAAAAAAAGCACCGACTCGGTGCC
NT374	TATGGATCCGGAAGAGCCAAAGTCAACACTTATAGAC
NT375	GTTGTAAAACGACGGCCAGTGCCAAGCTTC
NT376	TTAGCTAAAGATTTTAAACTTGGTATAATTGAATTGGAAGAGCCAAAGTCAAC
NT377	GTTGACTTTGGCTCTTCCAATTCAATTATACCAAGTTTAAAATCTTTAGCTAA
NT378	TTTATTATGCATCCTAGTACATATTATATAATTTAATTTGGAAGAGCCAAAGTCAACAC
NT379	GTGTTGACTTTGGCTCTTCCAAATTAAATTATATAATATGTACTAGGATGCATAATAAA
NT382	TCTTTTTTTTTAATTTTTGTGCTATTCTTTTTAACGGAAGAGCCAAAGTCAACACTT
NT383	AAGTGTTGACTTTGGCTCTTCCGTTAAAAAGAATAGCACAAAAATTAAAAAAAAAGA
NT384	CTTATATTGACAATCTAAGTATAATATTAAGGGAAGAGCCAAAGTCAACACTT
NT385	AAGTGTTGACTTTGGCTCTTCCCTTAATATTATACTTAGATTGTCAATATAAG
NT386	GAAAAGTATTTAAATAAGTGTCAATATTTTGTATTATTTAATTGGAAGAGCCAAAGTCAACACTT
NT387	AAGTGTTGACTTTGGCTCTTCCAATTAAATAATACAAAATATTGACACTTATTTAAATACTTTTC
NT388	TGATATTTTGATTTTTTATGATTAGAATCATCATGGAAGAGCCAAAGTCAACA
NT389	TGTTGACTTTGGCTCTTCCATGATGATTCTAATCATAAAAAATCAAAATATCA
NT390	CAAAGTTAACAGCAATGAAGTTTATAATAAATTGGAAGAGCCAAAGTCAA
NT391	TTGACTTTGGCTCTTCCAATTTATTATAAACTTCATTGCTGTTAACTTTG
NT392	AACTAAACTTTGAAAGCCTTGTTATAATATAAAATGGAAGAGCCAAAGTCAAC
NT393	GTTGACTTTGGCTCTTCCATTTTATATTATAACAAGGCTTTCAAAGTTTAGTT
NT394	CGAATTCTAAAGATCTTTGACAGCTAGCTCAGTCCTAGGTATAATACTAGTG
NT395	GATCCACTAGTATTATACCTAGGACTGAGCTAGCTGTCAAAGATCTTTAGAATTCGAGCT
NT402	ATTGAAAATTTAAATTTCTGACTT
NT403	TTTGAAAATTTAAATTTCTGACTT
NT404	AAGGAAAATTTAAATTTCTGACTT
NT405	CATGAAAATTTAAATTTCTGACTT
NT406	AATGAAAATTTAAATTTCTGACTT
NT409	CTTTTTTTTTAATTTTTGTGCTATTCTTTTTAACGAAAATTTAAATTTCTGACTTGTTTTAGAGCTAGAAATAGCAAGTTAAAATAAGG
NT410	CCTTATTTTAACTTGCTATTTCTAGCTCTAAAACAAGTCAGAAATTTAAATTTTCGTTAAAAAGAATAGCACAAAAATTAAAAAAAAAG
NT446	AATTGGCGCGCCAGGTTAATCTTCAATAACATG
NT449	GAGCGGCCGGAAACAGCTATGACATGATTACGAATTCG
NT593	CAACAGGGACACGGGCATTATTTACTAGTCACTATTTTAAATTCCAGGCGATAAAACC
NT594	TGATTTGAGTCAGCTAGGAGGTGACTAACCCGGGTAAAAAAACAAAAGATCCTTTAAAGG
NT595	CGCCTGGAATTTAAAATAGTGACTAGTAAATAATGCCCGTGTCCCTGTTGAATAGGG
NT596	GGATCTTTTGTTTTTTTACCCGGGTTAGTCACCTCCTAGCTGACTCAAATCAATGCG
NT611	AGTGAGTGTTATTAAGCATTCTTT
NT612	AACAAAGAATGCTTAATAACACTC
NT619	AGTGTTAATCTACCTAATATACCA
NT620	AACTGGTATATTAGGTAGATTAAC
NT654	CATGAGTGTTATTAAGCATTCTTT
NT655	CATGATATCTATTGCAACAACAGA
NT656	CATGTTAATCTACCTAATATACCA
NT669	GATAACAATTTCACACAGAATTCATTAAAGAAGAGAAATTACATATGGATAAGAAATAC
NT670	GTATTTCTTATCCATATGTAATTTCTCTTCTTTAATGAATTCTGTGTGAAATTGTTATC
NT671	GCTTTGTGAGCGGATAACAATTATACTAGATTCAATTGTGAGCGGATAACAATTTC
NT672	GAAATTGTTATCCGCTCACAATTGAATCTAGTATAATTGTTATCCGCTCACAAAGC
NT673	GCTTTGTGAGCGGATAACAATTATAATCGATTCAATTGTGAGCGGATAACAATTTCACAC
NT674	GTGTGAAATTGTTATCCGCTCACAATTGAATCGATTATAATTGTTATCCGCTCACAAAGC
NT675	GCTTTGTGAGCGGATAACAATTATACTCGATTCAATTGTGAGCGGATAACAATTTCACAC
NT676	GTGTGAAATTGTTATCCGCTCACAATTGAATCGAGTATAATTGTTATCCGCTCACAAAGC
NT677	GCTTTGTGAGCGGATAACAATTATAACAGATTCAATTGTGAGCGGATAACAATTTCACAC
NT678	GTGTGAAATTGTTATCCGCTCACAATTGAATCTGTTATAATTGTTATCCGCTCACAAAGC
NT680	AACTCTGTTGTTGCAATAGATATC
NT698	TATGACGTCATTAGAAAAACTCATCGAGCATCAAATGAAACTGC
NT699	TATGACGTCATTAGGTGGCGGTACTTGGGTCGATATCAAAG
NT700	TATGACGTCATTAACCTTCCCAAACATAACCACTAGG
NT701	TATGACGTCATTATTCTTTAGCTCTAGGTCTAGTACTAGGTCTTCTATTACC
NT702	TATGGATCCCAGCTTTTTTTTGAAGTGCCTGGCAGTAAGTTG
Primers used to confirm correct homologous recombination in strain CJW_Bb362	
NT424	GAGTAGTTAAGAGTTCTTCTGAAAG
NT425	CCTATAAAGATATATTGCCTTTAAGTG
NT591	GCGCCAGAGTTGTTTCTGAAACATGGC
NT592	GGTATCGGTCTGCGATTCCGACTCG
NT681	GCGATTGCAGTTGAAGCAGCATGTACTAATGTTCCAGC
NT682	CCCCAACTCTAAGTCCACTTTCAGTAATAGCTCGC
NT683	GGATGGTACTGAGGAATTATTGGTGAAACTAAATCG
NT684	GCTGGTTTTCGCATTCCTTCAGTAACATATTTGACC

a Where applicable, restriction enzyme sites are underlined in the primer sequence.

### DNA manipulations.

**(i) General methods.** Standard molecular biology techniques were used to generate the plasmids listed in Table 1. Oligonucleotide primers were purchased from Integrated DNA Technologies (IDT), and their sequences are provided in Table 3. PCR was performed using Platinum hot start PCR mastermix or Phusion high-fidelity DNA polymerase (Thermo Fisher Scientific). Restriction endonucleases were obtained from New England Biolabs or Thermo Fisher Scientific. Gel extraction was done using the PureLink quick gel extraction kit (Thermo Fisher Scientific). Gibson assembly mastermix was procured from New England Biolabs. Agilent’s QuikChange Lightning site-directed mutagenesis kit was used according to the manufacturer’s protocol. New England Biolabs ElectroLigase or quick ligation kits were used. Transformations of the cloning strains DH5α (Promega), NEB 5-alpha (New England Biolabs), or XL-10 Gold (Agilent) were done by electroporation or heat shock. Plasmids carrying the *dcas9-lacI* cassette were recovered and propagated in the NEB 5-alpha F′ *I^q^* strain (New England Biolabs). Cloning and/or propagation of plasmids containing the *dcas9-lacI* cassette in host E. coli strains that did not express *lacI^q^* often led to selection of clones that carried inactivating deletions within P*_pQE30_*, which abrogated *dcas9* expression in both E. coli and B. burgdorferi. This was likely due to high basal expression of *dcas9* in E. coli and associated nonspecific toxic effects. Plasmid DNA was isolated using a Zyppy plasmid miniprep kit (Zymo Research) or Qiagen plasmid midi kit. Correct DNA sequences of the relevant parts of the plasmids generated in this study were confirmed by Sanger sequencing at Quintarabio or at the Yale Keck Biotechnology Resource Laboratory.

**(ii) Generation of sgRNA cassettes.** The cloning template for an sgRNA cassette, modeled after a previous report (19), contains the following features: a promoter, a 500-bp filler sequence, a dCas9 handle region, and a transcriptional terminator sequence (Fig. 1A). To generate the P_syn_-sgRNA500 template cassette, a gene block (MS0, Table 3) containing the promoter P*_syn_*, the dCas9 handle region, and the transcriptional terminator, as well as regions homologous to the B. burgdorferi shuttle vectors, was synthesized at Integrated DNA Technologies. This gene block was Gibson assembled with the backbones of pBSV2 (78) and pBSV2G (8), which were obtained by PCR amplification using primers MS1 and MS2, respectively. The resulting plasmids were PCR-amplified using primers MS3 and MS4, and Gibson-assembled with an ∼500-bp filler DNA sequence. This filler was obtained by PCR amplification of the luciferase gene of pJSB252 (32) using primers MS5 and MS6.

To place sgRNA expression under the control of constitutive B. burgdorferi promoters (see Table S1 in the supplemental material), the following steps were taken. The sgRNA500 sequence was PCR amplified with primers NT374 and NT375, digested with BamHI and HindIII, and cloned into the BamHI and HindIII sites of shuttle vectors generated previously (6) that contain the following promoters between their SacI and BamHI sites: P*_flaB_*, P*_resT_*, P*_0026_*, P*_0031_*, P*_0526_*, or P*_0826_*. Next, the 5′ UTR contained within these promoter sequences (Table S1), as well as the BamHI site, were deleted by site-directed mutagenesis. The following primer pairs were used on the appropriate template: NT382 and NT383 generated the P*_flaBS_*-sgRNA500 cassette, NT378 and NT379 generated P*_resTS_*-sgRNA500, NT376 and NT377 generated P*_resTL_*-sgRNA500, NT392 and NT393 generated P*_0826S_*-sgRNA500, NT390 and NT391 generated P*_0826L_*-sgRNA500, NT384 and NT385 generated P*_0026_*-sgRNA500, NT388 and NT389 generated P*_0526_*-sgRNA500, and NT386 and NT387 generated P*_0031_*-sgRNA500.

To generate mature sgRNA cassettes targeting specific genes, the 500-bp filler was excised from plasmids containing the template sgRNA500 cassette by digestion with SapI, BspQI, or LguI. Pairs of primers (Tables 3 and 4) were annealed, generating a short double-stranded DNA (dsDNA) sequence with overhangs complementary to the overhangs generated by SapI, BspQI, or LguI digestion of the plasmid. The digested plasmid and annealed primers were ligated. To obtain pBSV2G_P_flaBS_-sgRNAflaB, site-directed mutagenesis was performed on pBSV2G_P_flaBS_-sgRNA500 using primers NT409 and NT410.

**TABLE 4:** Primer pairs used to generate the mature sgRNA cassettes^*a*^

Cassette	Forward primer	Reverse primer
P*_syn_*-sgRNAflaB	MS7	MS8
P*_syn_*-sgRNAftsI	NT611	NT612
P*_syn_*-sgRNAmreB^*b*^	MS9	NT680
P*_syn_*-sgRNArodA	NT619	NT620
P*_resTS_*-sgRNAflaB	NT403	MS8
P*_resTL_*-sgRNAflaB	NT402	MS8
P*_0826S_*-sgRNAflaB	NT406	MS8
P*_0826L_*-sgRNAflaB	NT402	MS8
P*_0026_*-sgRNAflaB	NT404	MS8
P*_0031_*-sgRNAflaB	NT402	MS8
P*_0526_*-sgRNAflaB	NT405	MS8
P*_0526_*-sgRNAftsI	NT654	NT612
P*_0526_*-sgRNAmreB	NT655	NT680
P*_0526_*-sgRNArodA	NT656	NT620

a The nucleotide sequences of these primers are provided in Table 3.

b While we lost the record of the primer sequences used to generate the P*_syn_*-sgRNAmreB cassette, primers MS9 and NT680 would allow its regeneration. The sequence of the generated cassette was confirmed to be correct by Sanger DNA sequencing.

### (iii) Choice of sgRNA target base-pairing sequence.

The coding sequence of a gene of interest, in the 5′ to 3′ orientation and including ∼100 bp upstream of the gene (to ensure that the 5′ UTR, if present, was included), was imported into the Geneious R10 software package. CRISPR sites were then identified using the “Find CRISPR sites” feature of the software. The required parameters were a 20-nucleotide base pairing region upstream of an NGG-3′ PAM site. CRISPR target sites were selected for further evaluation based on the following criteria: (i) they mapped within the target gene’s 5′ UTR or within its protein-coding region close to the translational start site, and (ii) their orientation was opposite that of the coding region, thus ensuring that the sgRNA targets the nontemplate strand (18, 19). Next, the NCBI webtool BLASTn was used to compare the CRISPR target site sequence against the complete B. burgdorferi B31 genome sequence (79) to rule out off-target binding. Primer pairs were then designed to encode sequences complementary to the CRISPR target sites, minus the PAM. A guanine base was added 5′ to the base-pairing sequence to ensure similar efficiency of transcription of the various sgRNAs and to account for the purine preference at the +1 position of the transcriptional start site (TSS) observed across several bacterial genomes (80, 81). Finally, the primers were designed to also generate, upon annealing, overhangs compatible with the SapI-digested plasmids containing template sgRNA cassettes.

### (iv) Transfer of sgRNA cassettes.

Among the shuttle vectors that do not contain the *dcas9-lacI* cassette, sgRNA cassettes were transferred as SacI/FspI restriction fragments. P*_syn_*-containing cassettes (e.g., P_syn_-sgRNAflaB) were inserted into pBbdCas9 vectors as AscI/EagI digests of PCR products generated using primers NT354 and NT355 and the appropriate template DNA. All other cassettes were inserted into pBbdCas9 vectors as AscI/EagI digests of PCR products generated using primers NT355 and NT449. The sgRNA cassettes were transferred among various pBbdCas9 vectors (see below) as either AscI/EagI or EagI/XmaI digests.

### (v) Updated sequence of pBSV2H.

During this work, we discovered that pBSV2H, which we generated as part of our previous study (6), contains a duplication of its dual rifampin-hygromycin B resistance cassette. This was confirmed by quality control tests performed at Addgene. We have therefore updated the sequence of the construct on Addgene’s product page (catalog no. 118229). All pBSV2H-derived plasmids generated in this study (Table 1) also contain this duplication. However, based on the normal behavior of the B. burgdorferi strains generated with these plasmids (see results obtained using chromosomal *dcas9* CRISPRi strains), we believe that the antibiotic cassette duplication does not affect the functionality of these vectors. Nevertheless, we generated a version of this shuttle vector that carries only one copy of the dual antibiotic resistance cassette, which we named pBSV2H_2 (Table 1).

### (vi) Generation of all-in-one CRISPRi shuttle vectors.

The luciferase gene of pJSB252 (32) was replaced with *dcas9* as follows. The pJSB252 backbone was PCR amplified with primers MS11 and MS12. The *dcas9* gene encoding the catalytically inactive protein (18) was PCR-amplified from plasmid pdCas9-bacteria using primers MS13 and MS14. The two fragments were Gibson assembled. Next, a silent mutation was introduced into the *lacI* gene to remove the SapI site. This was done by site-directed mutagenesis using NT352 and NT353, yielding plasmid pBbdCas9S. We note that the *dcas9* gene present in our constructs carries a mutation that results in a M1169L amino acid change, but *dcas9* remains functional despite this change. The *arr2* rifampin resistance gene was then PCR amplified from pBSV2B (6) using primers NT170 and NT446, digested with AscI and EagI, and cloned into the AscI/EagI backbone of pBbdCas9S to yield pBbdCas9S_arr2. P*_flgB_*-driven antibiotic resistance marker cassettes were generated as follows: primers NT698 and NT702 were used to PCR amplify the kanamycin cassette of pBSV2_2, NT699 and NT702 were used to amplify the gentamicin cassette of pBSV2G_2, NT700 and NT702 were used to amplify the blasticidin S cassette of pBSV2B, and NT701 and NT702 were used to amplify the hygromycin B cassette of pBSV2H. The resulting PCR products were digested with BamHI and AatII and ligated into the backbone of pBbdCas9S_arr2 obtained following sequential BglII and AatII digestion. This process generated the following plasmids: pBbdCas9K_arr2 (kanamycin resistant), pBbdCas9G_arr2 (gentamicin resistant), pBbdCas9B_arr2 (blasticidin S resistant), and pBbdCas9H_arr2 (hygromycin B resistant) (Table 1).

### (vii) Altered regulation of *dcas9* expression.

The following primer pairs were used to modify, by site-directed mutagenesis, the DNA region upstream of the *dcas9* coding sequence. NT669 and NT670 were used to mutate the ribosomal binding site, generating RBSmut constructs (Fig. 1H). The rest of the mutagenesis reactions modified the −10 region of the *dcas9* promoter, P*_pQE30_*, as follows: NT677 and NT678 were used to generate the −10TC construct, NT671 and NT672 for the −10AC1 construct, NT673 and NT674 for the −10AC2 construct, and NT675 and NT676 for the −10AC12 construct (Fig. 1H and Fig. S1F in the supplemental material).

### (viii) Suicide vector for chromosome integration of the *dcas9-lacI* cassette.

The following gene segments were assembled through a series of intermediate constructs. (i) The *aphI* gene of pCR2.1 TOPO was deleted by Gibson assembly using primers MS15 and MS16. The *bla* gene of the resulting backbone was deleted by site-directed mutagenesis using primers NT89 and NT90. The resulting backbone retains the E. coli origin of replication of pCR2.1 and its multicloning site. (ii) The antibiotic resistance cassette was assembled into this backbone by linking P*_flgB_* (flanked by SacII and NdeI sites), the *aphI* gene (flanked by NdeI and XmaI sites), and the *flaB* terminator, obtained by annealing primers NT350 and NT351 and inserting them between sites SpeI and XmaI. (iii) The sequence from nucleotide 474180 to nucleotide 476218 of the B31 chromosome was amplified with primers NT267 and NT268 and cloned as a SacI/SpeI fragment. (iv) The sequence from nucleotide 476251 to nucleotide 478279 of the B31 chromosome was amplified with primers NT269 and NT270 and cloned as a PstI/XhoI fragment. The backbone, antibiotic resistance cassette, and the two homology regions allow insertion by double crossover into the B31 chromosome between nucleotide 476218 and nucleotide 476251 in the intergenic region between genes *bb0456* and *bb0457*. This suicide vector sequence was amplified with primers NT593 and NT594, while the *dcas9-lacI* cassette was PCR amplified using primers NT595 and NT596 from pBbdCas9S_P_resTS_-sgRNAflaB. These two PCR products were Gibson assembled to yield pKIKan_idCas9_Chr_center.

### (ix) pBSV2_Psyn-mCherry^Bb^.

Primers NT394 and NT395 were annealed and ligated into the SacI/BamHI backbone of pBSV2_P_resT_-mCherry^Bb^ (6).

### RNA isolation and qRT-PCR.

Exponentially growing cultures of the CRISPRi strains were counted and diluted to 10^6^ cells/ml for next-day harvesting, or to 10^5^ cells/ml for harvesting on day 2. For situations in which IPTG induction caused growth defects, the culture induced for 2 days was also started at a density of 10^6^ cells/ml to ensure that similar amounts of RNA were obtained as in the noninduced culture at the time of harvest. Dilutions were carried out in 25 ml of BSK-II medium with or without 0.1 mM IPTG. At 24 or 48 h postinduction, bacteria were harvested by centrifugation of the 25-ml culture for 10 min at 4,300 × *g* and room temperature in a swinging bucket centrifuge. Supernatant was aspirated and the pellet was resuspended in 400 to 500 μl HN buffer (50 mM NaCl and 10 mM HEPES [pH 8.0]) (82). The suspension was then immediately added to 1 ml RNAprotect bacteria reagent (Qiagen), vortexed, incubated for 5 min at room temperature, and centrifuged at 10,000 × *g* and room temperature for 10 min. The supernatant was aspirated and the pellet was stored at −80°C until RNA could be extracted using an enzymatic lysis and proteinase K digestion method (protocol 4 in the RNAprotect bacteria reagent manual), followed by purification using the RNeasy minikit (Qiagen). A DNase digestion step was then performed using the Turbo DNA-free kit (Thermo Fisher Scientific), and purified RNA was stored at −80°C.

The Kapa SYBR Fast one-step qRT-PCR mastermix kit was used to quantify transcript levels. Reactions were done in duplicate or triplicate, using 10 ng total RNA per reaction. At least one additional reaction was performed for each sample without the addition of the reverse transcriptase mix. The quantification cycle (*C_q_*) values obtained from these control reactions confirmed that none of our results could be explained by DNA contamination of the RNA sample. The following protocol was used on a Bio-Rad iCycler: reverse transcription (5 min at 42°C); enzyme activation (3 min at 95°C); 40 cycles of annealing, extension, and data acquisition (3 sec at 95°C and 20 sec at 60°C); and a melt curve analysis (55 to 95°C in 0.5°C increments). The primers used to PCR amplify the various targets are provided in Table 5. The amount of each transcript of interest was normalized to the level of *recA* transcript in the same sample and then expressed relative to the level of target transcript in the control sample, as previously described (83).

**TABLE 5:** Primers used for qRT-PCR

Gene	Primer type	Sequence (5′ to 3′)	Reference or source
*flaB*	Forward	TTCAATCAGGTAACGGCACA	89
	Reverse	GACGCTTGAGACCCTGAAAG	89
*recA*	Forward	GTGGATCTATTGTATTAGATGAGGCTCTCG	90
	Reverse	GCCAAAGTTCTGCAACATTAACACCTAAAG	90
*dcas9*	Forward	AAGTAATGGGGCGGCATAAG	This study
	Reverse	TCTGGCCCTTTTGAGTTGTC	This study
*lacI*	Forward	CCTTGTTGCATTAGGCCATC	This study
	Reverse	TGTGCCATCCTGCTAATCTC	This study
*ftsI*	Forward	CGGAGAAACAAAAGGACTGC	This study
	Reverse	ATTTGAACCGCTGACACTCC	This study
*mreB*	Forward	TGTGATATTGGGGGTGGAAC	This study
	Reverse	AAATTCGTCACCACCAGTCC	This study
*rodA*	Forward	CCACGCTAATTATGTGCCATC	This study
	Reverse	CCAAAAACCCAAACTCTTCG	This study

### Microscopy.

Routine darkfield imaging of cultures was accomplished using a Nikon 40× 0.55 numerical aperture (NA) Ph2 phase-contrast air objective mounted on a Nikon Eclipse E600 microscope equipped with a darkfield condenser ring. Darkfield images and movies were acquired on a Nikon Eclipse Ti microscope equipped with a 40× 0.60 NA objective, a Nikon dry darkfield condenser (0.80 to 0.95 NA), and a Hamamatsu Orca-Flash4.0 v2 digital complementary metal oxide semiconductor (CMOS) camera. The same microscope was used to obtain phase contrast images using a 100× Plan Apo 1.45 NA Ph3 phase-contrast oil immersion objective and a Ph3 condenser ring. The microscope was controlled using the Nikon Elements software. Fluorescence imaging of strain CJW_Bb122 was done on a Nikon Eclipse Ti microscope with the following features: a 100× Plan Apo 1.40 NA Ph3 phase-contrast oil objective; a Hamamatsu Orca-Flash4.0 v2 CMOS camera; a Sola light engine (Lumencor); an mCherry/Texas Red fluorescence filter cube containing an ET560/40× excitation filter, a T585lpxr dichroic mirror, and an ET630/75m emission filter (Chroma); and Metamorph software (Molecular Devices). Images were processed using Nikon Elements Software, Metamorph software, or Fiji software (84).

### Image analysis.

Cell outlines were generated based on phase-contrast images using Oufti, our open-source analysis software package (58). The raw outlines were curated as follows: (i) outlines assigned to cells that crossed other cells, folded upon themselves, or were only partially present in the imaged frame, as well as outlines assigned to image components other than cells, were manually removed; (ii) outlines were manually extended to the tips of the cells where appropriate; (iii) outlines were manually added to cells whose geometry could support an outline but whose outlines were not generated automatically by Oufti; and (iv) when clear dips in the phase signal indicated an outer membrane bridge between two cytoplasmic cylinders that had separated as part of the cell division process, the two sides of the cell were treated as independent cellular units. In such cases, separate outlines generated by Oufti were left in place, while single outlines were manually split at the point at which the phase signal was observed to dip. Finally, all outlines were refined using the “Refine ALL” function of the Oufti software, and the fluorescence signal data were added to the outlines. Fluorescence quantification was done as previously described, using the MATLAB script addMeshtoCellList.m and the function CalculateFluorPerCell.m (6). Cell lengths were extracted using the MATLAB script get_um_lengths.m (85), and values below 1 μm were excluded from the analysis.

### Cryo-electron tomography and three-dimensional visualization.

B. burgdorferi cultures growing exponentially in BSK-II medium to no more than 5 × 10^7^ cells/ml were pelleted for 10 min at 3,000 × *g* at room temperature. The pellet was gently resuspended in a small volume (50 to 100 μl) of BSK-H (catalog no. B8291; Sigma-Aldrich). The cell suspension was then mixed with 10-nm colloidal gold fiducial markers. Aliquots (5 μl) of cell suspension were deposited on holey carbon electron microscopy grids (200 mesh, R2/1; Quantifoil), which had been freshly glow discharged for ∼30 s. The grids were then blotted with filter paper and rapidly frozen in liquid ethane using a homemade gravity-driven plunger apparatus. The frozen-hydrated specimens were transferred to a 300 kV Titan Krios electron microscope (Thermo Fisher Scientific) equipped with a K2 direct electron detector and energy filter (Gatan). SerialEM (86) was used to collect single-axis tilt series around −5 μm defocus, with a cumulative dose of ∼70 electrons (e^−^)/Å covering angles from −51° to 51° with a 3° tilt step. Images were acquired at 26,000× magnification with an effective pixel size of 5.457 Å at the specimen level. All recorded images were first drift corrected using the MotionCor2 software (87) and then stacked by the software package IMOD (88). In total, 11 tilt series were aligned and reconstructed using IMOD. Three-dimensional models of the flagella and cells were manually segmented and visualized by IMOD.

### Data availability.

Plasmids generated in this study (and their sequences) are available through Addgene under the accession numbers listed in Table 1. Strain CJW_Bb362 is available through ATCC’s BEI Resources collection (item no. NR-53512). Reasonable requests for all other B. burgdorferi strains generated in this study shall be honored by the Jacobs-Wagner lab. The MATLAB code used to process cell fluorescence data can be downloaded from GitHub (85). MATLAB code, including dependencies, is provided at GitHub (https://github.com/JacobsWagnerLab/published).

## Supplementary Material

Supplemental file 1

Supplemental file 2

Supplemental file 3

Supplemental file 4

Supplemental file 5

Supplemental file 6

Supplemental file 7

Supplemental file 8

Supplemental file 9

## References

[1] Radolf JD, Caimano MJ, Stevenson B, Hu LT 2012 Of ticks, mice and men: understanding the dual-host lifestyle of Lyme disease spirochaetes. Nat Rev Microbiol 10:87–99. doi:10.1038/nrmicro2714. 22230951 PMC3313462

[2] Mead PS 2015 Epidemiology of Lyme disease. Infect Dis Clin North Am 29:187–210. doi:10.1016/j.idc.2015.02.010. 25999219

[3] Steere AC, Strle F, Wormser GP, Hu LT, Branda JA, Hovius JW, Li X, Mead PS 2016 Lyme borreliosis. Nat Rev Dis Primers 2:16090. doi:10.1038/nrdp.2016.90. 27976670 PMC5539539

[4] Samuels DS, Drecktrah D, Hall LS 2018 Genetic transformation and complementation. Methods Mol Biol 1690:183–200. doi:10.1007/978-1-4939-7383-5_15. 29032546 PMC5806694

[5] Drecktrah D, Samuels DS 2018 Genetic manipulation of *Borrelia* spp. Curr Top Microbiol Immunol 415:113–140. doi:10.1007/82_2017_51. 28918538 PMC5857249

[6] Takacs CN, Kloos ZA, Scott M, Rosa PA, Jacobs-Wagner C 2018 Fluorescent proteins, promoters, and selectable markers for applications in the Lyme disease spirochete *Borrelia burgdorferi*. Appl Environ Microbiol 84:e01824-18. doi:10.1128/AEM.01824-18. 30315081 PMC6275353

[7] Bono JL, Elias AF, Kupko JJ, 3rd, Stevenson B, Tilly K, Rosa P 2000 Efficient targeted mutagenesis in *Borrelia burgdorferi*. J Bacteriol 182:2445–2452. doi:10.1128/jb.182.9.2445-2452.2000. 10762244 PMC111306

[8] Elias AF, Bono JL, Kupko JJ, 3rd, Stewart PE, Krum JG, Rosa PA 2003 New antibiotic resistance cassettes suitable for genetic studies in *Borrelia burgdorferi*. J Mol Microbiol Biotechnol 6:29–40. doi:10.1159/000073406. 14593251

[9] Tilly K, Elias AF, Bono JL, Stewart P, Rosa P 2000 DNA exchange and insertional inactivation in spirochetes. J Mol Microbiol Biotechnol 2:433–442. 11075915

[10] Criswell D, Tobiason VL, Lodmell JS, Samuels DS 2006 Mutations conferring aminoglycoside and spectinomycin resistance in *Borrelia burgdorferi*. Antimicrob Agents Chemother 50:445–452. doi:10.1128/AAC.50.2.445-452.2006. 16436695 PMC1366916

[11] Barbour AG 1984 Isolation and cultivation of Lyme disease spirochetes. Yale J Biol Med 57:521–525. 6393604 PMC2589996

[12] Elias AF, Stewart PE, Grimm D, Caimano MJ, Eggers CH, Tilly K, Bono JL, Akins DR, Radolf JD, Schwan TG, Rosa P 2002 Clonal polymorphism of *Borrelia burgdorferi* strain B31 MI: implications for mutagenesis in an infectious strain background. Infect Immun 70:2139–2150. doi:10.1128/iai.70.4.2139-2150.2002. 11895980 PMC127854

[13] Zuckert WR 2007 Laboratory maintenance of *Borrelia burgdorferi*. Curr Protoc Microbiol Chapter 12:Unit 12C.1. doi:10.1002/9780471729259.mc12c01s4. 18770608

[14] Jutras BL, Chenail AM, Stevenson B 2013 Changes in bacterial growth rate govern expression of the *Borrelia burgdorferi* OspC and Erp infection-associated surface proteins. J Bacteriol 195:757–764. doi:10.1128/JB.01956-12. 23222718 PMC3562092

[15] Elias A, Bono JL, Tilly K, Rosa P 1998 Growth of infectious and non-infectious *B. burgdorferi* at different salt concentrations. Wien Klin Wochenschr 110:863–865. 10048166

[16] Heroldova M, Nemec M, Hubalek Z 1998 Growth parameters of *Borrelia burgdorferi* sensu stricto at various temperatures. Zentralbl Bakteriol 288:451–455. doi:10.1016/S0934-8840(98)80058-3. 9987182

[17] Latham JI, Blevins JS 2018 Generation of conditional mutants in *Borrelia burgdorferi*. Methods Mol Biol 1690:225–239. doi:10.1007/978-1-4939-7383-5_17. 29032548

[18] Qi LS, Larson MH, Gilbert LA, Doudna JA, Weissman JS, Arkin AP, Lim WA 2013 Repurposing CRISPR as an RNA-guided platform for sequence-specific control of gene expression. Cell 152:1173–1183. doi:10.1016/j.cell.2013.02.022. 23452860 PMC3664290

[19] Larson MH, Gilbert LA, Wang X, Lim WA, Weissman JS, Qi LS 2013 CRISPR interference (CRISPRi) for sequence-specific control of gene expression. Nat Protoc 8:2180–2196. doi:10.1038/nprot.2013.132. 24136345 PMC3922765

[20] Jinek M, Chylinski K, Fonfara I, Hauer M, Doudna JA, Charpentier E 2012 A programmable dual-RNA-guided DNA endonuclease in adaptive bacterial immunity. Science 337:816–821. doi:10.1126/science.1225829. 22745249 PMC6286148

[21] Koonin EV, Makarova KS, Zhang F 2017 Diversity, classification and evolution of CRISPR-Cas systems. Curr Opin Microbiol 37:67–78. doi:10.1016/j.mib.2017.05.008. 28605718 PMC5776717

[22] Jones DL, Leroy P, Unoson C, Fange D, Ćurić V, Lawson MJ, Elf J 2017 Kinetics of dCas9 target search in *Escherichia coli*. Science 357:1420–1424. doi:10.1126/science.aah7084. 28963258 PMC6150439

[23] Gilbert LA, Larson MH, Morsut L, Liu Z, Brar GA, Torres SE, Stern-Ginossar N, Brandman O, Whitehead EH, Doudna JA, Lim WA, Weissman JS, Qi LS 2013 CRISPR-mediated modular RNA-guided regulation of transcription in eukaryotes. Cell 154:442–451. doi:10.1016/j.cell.2013.06.044. 23849981 PMC3770145

[24] Peters JM, Colavin A, Shi H, Czarny TL, Larson MH, Wong S, Hawkins JS, Lu CHS, Koo BM, Marta E, Shiver AL, Whitehead EH, Weissman JS, Brown ED, Qi LS, Huang KC, Gross CA 2016 A comprehensive, CRISPR-based functional analysis of essential genes in bacteria. Cell 165:1493–1506. doi:10.1016/j.cell.2016.05.003. 27238023 PMC4894308

[25] Westbrook AW, Moo-Young M, Chou CP 2016 Development of a CRISPR-Cas9 tool kit for comprehensive engineering of *Bacillus subtilis*. Appl Environ Microbiol 82:4876–4895. doi:10.1128/AEM.01159-16. 27260361 PMC4968543

[26] Irnov I, Wang Z, Jannetty ND, Bustamante JA, Rhee KY, Jacobs-Wagner C 2017 Crosstalk between the tricarboxylic acid cycle and peptidoglycan synthesis in *Caulobacter crescentus* through the homeostatic control of alpha-ketoglutarate. PLoS Genet 13:e1006978. doi:10.1371/journal.pgen.1006978. 28827812 PMC5578688

[27] Guzzo M, Castro LK, Reisch CR, Guo MS, Laub MT 2020 A CRISPR interference system for efficient and rapid gene knockdown in *Caulobacter crescentus*. mBio 11:e02415-19. doi:10.1128/mBio.02415-19. 31937638 PMC6960281

[28] Rock JM, Hopkins FF, Chavez A, Diallo M, Chase MR, Gerrick ER, Pritchard JR, Church GM, Rubin EJ, Sassetti CM, Schnappinger D, Fortune SM 2017 Programmable transcriptional repression in mycobacteria using an orthogonal CRISPR interference platform. Nat Microbiol 2:16274. doi:10.1038/nmicrobiol.2016.274. 28165460 PMC5302332

[29] Singh AK, Carette X, Potluri LP, Sharp JD, Xu R, Prisic S, Husson RN 2016 Investigating essential gene function in *Mycobacterium tuberculosis* using an efficient CRISPR interference system. Nucleic Acids Res 44:e143. doi:10.1093/nar/gkw625. 27407107 PMC5062980

[30] Choudhary E, Lunge A, Agarwal N 2016 Strategies of genome editing in mycobacteria: achievements and challenges. Tuberculosis (Edinb) 98:132–138. doi:10.1016/j.tube.2016.03.005. 27156629

[31] Fernandes LGV, Guaman LP, Vasconcellos SA, Heinemann MB, Picardeau M, Nascimento A 2019 Gene silencing based on RNA-guided catalytically inactive Cas9 (dCas9): a new tool for genetic engineering in *Leptospira*. Sci Rep 9:1839. doi:10.1038/s41598-018-37949-x. 30755626 PMC6372684

[32] Blevins JS, Revel AT, Smith AH, Bachlani GN, Norgard MV 2007 Adaptation of a luciferase gene reporter and *lac* expression system to *Borrelia burgdorferi*. Appl Environ Microbiol 73:1501–1513. doi:10.1128/AEM.02454-06. 17220265 PMC1828772

[33] Rego RO, Bestor A, Rosa PA 2011 Defining the plasmid-borne restriction-modification systems of the Lyme disease spirochete *Borrelia burgdorferi*. J Bacteriol 193:1161–1171. doi:10.1128/JB.01176-10. 21193609 PMC3067601

[34] Kasumba IN, Bestor A, Tilly K, Rosa PA 2015 Use of an endogenous plasmid locus for stable *in trans* complementation in *Borrelia burgdorferi*. Appl Environ Microbiol 81:1038–1046. doi:10.1128/AEM.03657-14. 25452278 PMC4292500

[35] Tilly K, Krum JG, Bestor A, Jewett MW, Grimm D, Bueschel D, Byram R, Dorward D, Vanraden MJ, Stewart P, Rosa P 2006 *Borrelia burgdorferi* OspC protein required exclusively in a crucial early stage of mammalian infection. Infect Immun 74:3554–3564. doi:10.1128/IAI.01950-05. 16714588 PMC1479285

[36] Beaurepaire C, Chaconas G 2007 Topology-dependent transcription in linear and circular plasmids of the segmented genome of *Borrelia burgdorferi*. Mol Microbiol 63:443–453. doi:10.1111/j.1365-2958.2006.05533.x. 17241200

[37] Alverson J, Bundle SF, Sohaskey CD, Lybecker MC, Samuels DS 2003 Transcriptional regulation of the *ospAB* and *ospC* promoters from *Borrelia burgdorferi*. Mol Microbiol 48:1665–1677. doi:10.1046/j.1365-2958.2003.03537.x. 12791146

[38] Alverson J, Samuels DS 2002 *groEL* expression in *gyrB* mutants of *Borrelia burgdorferi*. J Bacteriol 184:6069–6072. doi:10.1128/jb.183.21.6069-6072.2002. 12374843 PMC135381

[39] Deana A, Belasco JG 2005 Lost in translation: the influence of ribosomes on bacterial mRNA decay. Genes Dev 19:2526–2533. doi:10.1101/gad.1348805. 16264189

[40] Cho S, Choe D, Lee E, Kim SC, Palsson B, Cho BK 2018 High-level dCas9 expression induces abnormal cell morphology in *Escherichia coli*. ACS Synth Biol 7:1085–1094. doi:10.1021/acssynbio.7b00462. 29544049

[41] Motaleb MA, Corum L, Bono JL, Elias AF, Rosa P, Samuels DS, Charon NW 2000 *Borrelia burgdorferi* periplasmic flagella have both skeletal and motility functions. Proc Natl Acad Sci U S A 97:10899–10904. doi:10.1073/pnas.200221797. 10995478 PMC27121

[42] Goldstein SF, Charon NW, Kreiling JA 1994 *Borrelia burgdorferi* swims with a planar waveform similar to that of eukaryotic flagella. Proc Natl Acad Sci U S A 91:3433–3437. doi:10.1073/pnas.91.8.3433. 8159765 PMC43591

[43] Typas A, Banzhaf M, Gross CA, Vollmer W 2011 From the regulation of peptidoglycan synthesis to bacterial growth and morphology. Nat Rev Microbiol 10:123–136. doi:10.1038/nrmicro2677. 22203377 PMC5433867

[44] Botta GA, Park JT 1981 Evidence for involvement of penicillin-binding protein 3 in murein synthesis during septation but not during cell elongation. J Bacteriol 145:333–340. doi:10.1128/JB.145.1.333-340.1981. 6450748 PMC217277

[45] Spratt BG 1977 Temperature-sensitive cell division mutants of *Escherichia coli* with thermolabile penicillin-binding proteins. J Bacteriol 131:293–305. doi:10.1128/JB.131.1.293-305.1977. 326764 PMC235422

[46] Tamura T, Suzuki H, Nishimura Y, Mizoguchi J, Hirota Y 1980 On the process of cellular division in *Escherichia coli*: isolation and characterization of penicillin-binding proteins 1a, 1b, and 3. Proc Natl Acad Sci U S A 77:4499–4503. doi:10.1073/pnas.77.8.4499. 7001458 PMC349871

[47] Meeske AJ, Riley EP, Robins WP, Uehara T, Mekalanos JJ, Kahne D, Walker S, Kruse AC, Bernhardt TG, Rudner DZ 2016 SEDS proteins are a widespread family of bacterial cell wall polymerases. Nature 537:634–638. doi:10.1038/nature19331. 27525505 PMC5161649

[48] Shi H, Bratton BP, Gitai Z, Huang KC 2018 How to build a bacterial cell: MreB as the foreman of *E. coli* construction. Cell 172:1294–1305. doi:10.1016/j.cell.2018.02.050. 29522748 PMC5846203

[49] Matsuzawa H, Hayakawa K, Sato T, Imahori K 1973 Characterization and genetic analysis of a mutant of *Escherichia coli* K-12 with rounded morphology. J Bacteriol 115:436–442. doi:10.1128/JB.115.1.436-442.1973. 4577747 PMC246256

[50] Bendezu FO, de Boer PA 2008 Conditional lethality, division defects, membrane involution, and endocytosis in *mre* and *mrd* shape mutants of *Escherichia coli*. J Bacteriol 190:1792–1811. doi:10.1128/JB.01322-07. 17993535 PMC2258658

[51] Wagner JK, Galvani CD, Brun YV 2005 *Caulobacter crescentus* requires RodA and MreB for stalk synthesis and prevention of ectopic pole formation. J Bacteriol 187:544–553. doi:10.1128/JB.187.2.544-553.2005. 15629926 PMC543564

[52] Wachi M, Matsuhashi M 1989 Negative control of cell division by *mreB*, a gene that functions in determining the rod shape of *Escherichia coli* cells. J Bacteriol 171:3123–3127. doi:10.1128/jb.171.6.3123-3127.1989. 2656641 PMC210024

[53] Figge RM, Divakaruni AV, Gober JW 2004 MreB, the cell shape-determining bacterial actin homologue, co-ordinates cell wall morphogenesis in *Caulobacter crescentus*. Mol Microbiol 51:1321–1332. doi:10.1111/j.1365-2958.2003.03936.x. 14982627

[54] Arnold WK, Savage CR, Brissette CA, Seshu J, Livny J, Stevenson B 2016 RNA-seq of *Borrelia burgdorferi* in multiple phases of growth reveals insights into the dynamics of gene expression, transcriptome architecture, and noncoding RNAs. PLoS One 11:e0164165. doi:10.1371/journal.pone.0164165. 27706236 PMC5051733

[55] Hovind-Hougen K 1984 Ultrastructure of spirochetes isolated from *Ixodes ricinus* and *Ixodes dammini*. Yale J Biol Med 57:543–548. 6516456 PMC2590005

[56] Kudryashev M, Cyrklaff M, Baumeister W, Simon MM, Wallich R, Frischknecht F 2009 Comparative cryo-electron tomography of pathogenic Lyme disease spirochetes. Mol Microbiol 71:1415–1434. doi:10.1111/j.1365-2958.2009.06613.x. 19210619

[57] Zhang K, He J, Cantalano C, Guo Y, Liu J, Li C 2020 FlhF regulates the number and configuration of periplasmic flagella in *Borrelia burgdorferi*. Mol Microbiol 113:1122–1139. doi:10.1111/mmi.14482. 32039533 PMC8085991

[58] Paintdakhi A, Parry B, Campos M, Irnov I, Elf J, Surovtsev I, Jacobs-Wagner C 2016 Oufti: an integrated software package for high-accuracy, high-throughput quantitative microscopy analysis. Mol Microbiol 99:767–777. doi:10.1111/mmi.13264. 26538279 PMC4752901

[59] Jutras BL, Scott M, Parry B, Biboy J, Gray J, Vollmer W, Jacobs-Wagner C 2016 Lyme disease and relapsing fever *Borrelia* elongate through zones of peptidoglycan synthesis that mark division sites of daughter cells. Proc Natl Acad Sci U S A 113:9162–9170. doi:10.1073/pnas.1610805113. 27506799 PMC4995948

[60] Hinnebusch J, Barbour AG 1992 Linear- and circular-plasmid copy numbers in *Borrelia burgdorferi*. J Bacteriol 174:5251–5257. doi:10.1128/jb.174.16.5251-5257.1992. 1644750 PMC206359

[61] Lin T, Gao L, Zhang C, Odeh E, Jacobs MB, Coutte L, Chaconas G, Philipp MT, Norris SJ 2012 Analysis of an ordered, comprehensive STM mutant library in infectious *Borrelia burgdorferi*: insights into the genes required for mouse infectivity. PLoS One 7:e47532. doi:10.1371/journal.pone.0047532. 23133514 PMC3485029

[62] Gitai Z, Dye NA, Reisenauer A, Wachi M, Shapiro L 2005 MreB actin-mediated segregation of a specific region of a bacterial chromosome. Cell 120:329–341. doi:10.1016/j.cell.2005.01.007. 15707892

[63] Takacs CN, Poggio S, Charbon G, Pucheault M, Vollmer W, Jacobs-Wagner C 2010 MreB drives *de novo* rod morphogenesis in *Caulobacter crescentus* via remodeling of the cell wall. J Bacteriol 192:1671–1684. doi:10.1128/JB.01311-09. 20023035 PMC2832515

[64] Iwai N, Nagai K, Wachi M 2002 Novel *S*-benzylisothiourea compound that induces spherical cells in *Escherichia coli* probably by acting on a rod-shape-determining protein(s) other than penicillin-binding protein 2. Biosci Biotechnol Biochem 66:2658–2662. doi:10.1271/bbb.66.2658. 12596863

[65] Iwai N, Ebata T, Nagura H, Kitazume T, Nagai K, Wachi M 2004 Structure-activity relationship of *S*-benzylisothiourea derivatives to induce spherical cells in *Escherichia coli*. Biosci Biotechnol Biochem 68:2265–2269. doi:10.1271/bbb.68.2265. 15564663

[66] Iwai N, Fujii T, Nagura H, Wachi M, Kitazume T 2007 Structure-activity relationship study of the bacterial actin-like protein MreB inhibitors: effects of substitution of benzyl group in S-benzylisothiourea. Biosci Biotechnol Biochem 71:246–248. doi:10.1271/bbb.60443. 17213642

[67] Alekshun M, Kashlev M, Schwartz I 1997 Molecular cloning and characterization of *Borrelia burgdorferi rpoB*. Gene 186:227–235. doi:10.1016/s0378-1119(96)00714-7. 9074501

[68] Jiang S, Gilpin ME, Attia M, Ting YL, Berti PJ 2011 Lyme disease enolpyruvyl-UDP-GlcNAc synthase: fosfomycin-resistant MurA from *Borrelia burgdorferi*, a fosfomycin-sensitive mutant, and the catalytic role of the active site Asp. Biochemistry 50:2205–2212. doi:10.1021/bi1017842. 21294548

[69] Ohta N, Ninfa AJ, Allaire A, Kulick L, Newton A 1997 Identification, characterization, and chromosomal organization of cell division cycle genes in *Caulobacter crescentus*. J Bacteriol 179:2169–2180. doi:10.1128/jb.179.7.2169-2180.1997. 9079901 PMC178952

[70] Slamti L, de Pedro MA, Guichet E, Picardeau M 2011 Deciphering morphological determinants of the helix-shaped *Leptospira*. J Bacteriol 193:6266–6275. doi:10.1128/JB.05695-11. 21926230 PMC3209227

[71] Jones LJ, Carballido-Lopez R, Errington J 2001 Control of cell shape in bacteria: helical, actin-like filaments in *Bacillus subtilis*. Cell 104:913–922. doi:10.1016/s0092-8674(01)00287-2. 11290328

[72] Casadaban MJ, Cohen SN 1980 Analysis of gene control signals by DNA fusion and cloning in *Escherichia coli*. J Mol Biol 138:179–207. doi:10.1016/0022-2836(80)90283-1. 6997493

[73] Frank KL, Bundle SF, Kresge ME, Eggers CH, Samuels DS 2003 *aadA* confers streptomycin resistance in *Borrelia burgdorferi*. J Bacteriol 185:6723–6727. doi:10.1128/jb.185.22.6723-6727.2003. 14594849 PMC262111

[74] Samuels DS 1995 Electrotransformation of the spirochete *Borrelia burgdorferi*. Methods Mol Biol 47:253–259. doi:10.1385/0-89603-310-4:253. 7550741 PMC5815860

[75] Casjens S, van Vugt R, Tilly K, Rosa PA, Stevenson B 1997 Homology throughout the multiple 32-kilobase circular plasmids present in Lyme disease spirochetes. J Bacteriol 179:217–227. doi:10.1128/jb.179.1.217-227.1997. 8982001 PMC178682

[76] Green MR, Sambrook J 2016 Precipitation of DNA with ethanol. Cold Spring Harb Protoc 2016:pdb.prot093377–1120. doi:10.1101/pdb.prot093377. 27934690

[77] Bunikis I, Kutschan-Bunikis S, Bonde M, Bergstrom S 2011 Multiplex PCR as a tool for validating plasmid content of *Borrelia burgdorferi*. J Microbiol Methods 86:243–247. doi:10.1016/j.mimet.2011.05.004. 21605603

[78] Stewart PE, Thalken R, Bono JL, Rosa P 2001 Isolation of a circular plasmid region sufficient for autonomous replication and transformation of infectious *Borrelia burgdorferi*. Mol Microbiol 39:714–721. doi:10.1046/j.1365-2958.2001.02256.x. 11169111

[79] Fraser CM, Casjens S, Huang WM, Sutton GG, Clayton R, Lathigra R, White O, Ketchum KA, Dodson R, Hickey EK, Gwinn M, Dougherty B, Tomb JF, Fleischmann RD, Richardson D, Peterson J, Kerlavage AR, Quackenbush J, Salzberg S, Hanson M, van Vugt R, Palmer N, Adams MD, Gocayne J, Weidman J, Utterback T, Watthey L, McDonald L, Artiach P, Bowman C, Garland S, Fuji C, Cotton MD, Horst K, Roberts K, Hatch B, Smith HO, Venter JC 1997 Genomic sequence of a Lyme disease spirochaete, *Borrelia burgdorferi*. Nature 390:580–586. doi:10.1038/37551. 9403685

[80] Kim D, Hong JS, Qiu Y, Nagarajan H, Seo JH, Cho BK, Tsai SF, Palsson BO 2012 Comparative analysis of regulatory elements between *Escherichia coli* and *Klebsiella pneumoniae* by genome-wide transcription start site profiling. PLoS Genet 8:e1002867. doi:10.1371/journal.pgen.1002867. 22912590 PMC3415461

[81] Prados J, Linder P, Redder P 2016 TSS-EMOTE, a refined protocol for a more complete and less biased global mapping of transcription start sites in bacterial pathogens. BMC Genomics 17:849. doi:10.1186/s12864-016-3211-3. 27806702 PMC5094136

[82] Nowalk AJ, Gilmore RD, Jr, Carroll JA 2006 Serologic proteome analysis of *Borrelia burgdorferi* membrane-associated proteins. Infect Immun 74:3864–3873. doi:10.1128/IAI.00189-06. 16790758 PMC1489744

[83] Livak KJ, Schmittgen TD 2001 Analysis of relative gene expression data using real-time quantitative PCR and the 2^−ΔΔ^*^C_T_^* method. Methods 25:402–408. doi:10.1006/meth.2001.1262. 11846609

[84] Schindelin J, Arganda-Carreras I, Frise E, Kaynig V, Longair M, Pietzsch T, Preibisch S, Rueden C, Saalfeld S, Schmid B, Tinevez JY, White DJ, Hartenstein V, Eliceiri K, Tomancak P, Cardona A 2012 Fiji: an open-source platform for biological-image analysis. Nat Methods 9:676–682. doi:10.1038/nmeth.2019. 22743772 PMC3855844

[85] Takacs CN, Scott M, Chang Y, Kloos ZA, Irnov I, Rosa PA, Liu J, Jacobs-Wagner C 2020 Code for “A CRISPR interference platform for selective downregulation of gene expression in *Borrelia burgdorferi*.” GitHub Code Repository. https://github.com/JacobsWagnerLab/published/tree/master/Takacs_2020_CRISPRi. 10.1128/AEM.02519-20 PMC7851697 33257311

[86] Mastronarde DN 2005 Automated electron microscope tomography using robust prediction of specimen movements. J Struct Biol 152:36–51. doi:10.1016/j.jsb.2005.07.007. 16182563

[87] Zheng SQ, Palovcak E, Armache JP, Verba KA, Cheng Y, Agard DA 2017 MotionCor2: anisotropic correction of beam-induced motion for improved cryo-electron microscopy. Nat Methods 14:331–332. doi:10.1038/nmeth.4193. 28250466 PMC5494038

[88] Kremer JR, Mastronarde DN, McIntosh JR 1996 Computer visualization of three-dimensional image data using IMOD. J Struct Biol 116:71–76. doi:10.1006/jsbi.1996.0013. 8742726

[89] Narasimhan S, Sukumaran B, Bozdogan U, Thomas V, Liang X, DePonte K, Marcantonio N, Koski RA, Anderson JF, Kantor F, Fikrig E 2007 A tick antioxidant facilitates the Lyme disease agent's successful migration from the mammalian host to the arthropod vector. Cell Host Microbe 2:7–18. doi:10.1016/j.chom.2007.06.001. 18005713 PMC2699493

[90] Morrison TB, Ma Y, Weis JH, Weis JJ 1999 Rapid and sensitive quantification of *Borrelia burgdorferi*-infected mouse tissues by continuous fluorescent monitoring of PCR. J Clin Microbiol 37:987–992. doi:10.1128/JCM.37.4.987-992.1999. 10074514 PMC88637

