# Supplementary material for: A CRISPR Interference Platform for Selective Downregulation of Gene Expression in Borrelia burgdorferi
Source: Appl Environ Microbiol. 2021 Jan 29;87(4):e02519-20. doi: 10.1128/AEM.02519-20 (PMC7851697; doi:10.1128/AEM.02519-20)
Supplement: Supplemental file 1 [file AEM.02519-20-s0001.pdf]

A CRISPR interference platform for selective downregulation of gene expression in *Borrelia burgdorferi*.

Constantin N. Takacs, Molly Scott, Yunjie Chang, Zachary A. Kloos, Irnov Irnov, Patricia A. Rosa, Jun Liu, and Christine Jacobs-Wagner

SUPPLEMENTAL MATERIAL:

Supplemental text……………………………………………………………………………..S2
Supplemental tables…………………………………………………………………………..S3
Supplemental figures……………………………………………………………………….S5
Supplemental movie legends……………………………………………………………….S12
Supplemental references……………………………………………………………………S13

SUPPLEMENTAL TEXT

Pharmacologic attempts to study MreB function in *B. burgdorferi*.

Specific small-molecule inhibitors can be valuable tools in biological investigations. For instance, the compounds A22 and MP265 inhibit the bacterial actin homolog, MreB, causing cell rounding of *E. coli* and other rod-shaped bacteria (1-5). A22 is active in the related spirochete *Leptospira biflexa*, where it causes cell bulging (6). However, the effects of MreB inhibitors on *B. burgdorferi* morphology have not been reported.

When we exposed *B. burgdorferi* strain K2 for two days (about seven generations) to MP265 at 50 μM, the typical dose of MreB inhibitor used in other bacteria (1, 2, 6), we observed no detectable morphological changes (Fig. S7A). Increasing the MP265 dose to 500 μM or treating *B. burgdorferi* with 500 μM A22 also had no apparent effect on cell morphology (Fig. S7A). Both A22 and MP265 were active in BSK-II medium as 50 μM of either drug induced cell rounding in BSK-II-grown cells of the *E. coli* strain MC1000 (Fig. S7B). We also measured the effects of A22 and MP265 on *B. burgdorferi* growth. The occurrence of cell growth is an important consideration, as cell rounding or bulging phenotypes associated with MreB inactivation are growth-dependent (1). A22 did not affect the growth of *B. burgdorferi* when used at 50 or 500 μM, nor did MP265 when used at 50 μM (Fig. S7C). While 500 μM MP265 slightly reduced the growth of our strain (Fig. S7C), a significant amount of growth (about four generations) still occurred during the treatment. Thus, the lack of cell morphology defects in response of A22 or MP265 treatment cannot be attributed to a growth arrest. Our results therefore indicate that *B. burgdorferi* appears to be resistant to chemical inhibition of MreB by A22 or MP265.

SUPPLEMENTAL TABLES

Table S1. Promoters used to drive sgRNA expression in *B. burgdorferi*

Promoter name	Native gene	Strand	START codon position[a]	TSS position[b]	Reference for TSS	Promoter coordinates[a] 5' end	3' end
P_{syn}	None (synthetic promoter)	-	-	-	-	-	-
P_{flaBS}	*bb0147* (*flaB*)	reverse	148659	-56	(7, 8)	149013	148716
P_{resTL}	*bbb03* (*resT*)	reverse	2186	-3/-4	(8, 9)	2371	2190
P_{resTS}				-78	(8)	2371	2265
P_{0826L}	*bb0826* (*hyp*)	reverse	870023	0	(8)	870235	870024
P_{0826S}				-52/-54	(8)	870235	870076
P_{0526}	*bb0526* (*hyp*)	forward	535704	0	(8)	535523	535703
P_{0031}	*bb0031* (*lepB*)	reverse	29472	-14	(8, 9)	29670	29487
P_{0026}	*bb0026* (*folD2*)	reverse	25623	-9	(8, 9)	25752	25633

[a] Nucleotide positions within the chromosome (RefSeq NC_001318.1) or cp26 (for *resT*, RefSeq NC_001903.1). Records were last accessed on April 30, 2020;
[b] Nucleotide position relative to the first base of the START codon.

Table S2. Morphology and growth phenotypes observed in the generated CRISPRi strains in the absence of IPTG induction of *dcas9* expression.

CRISPRi version	Gene targeted	P_{pQE30} mutation[a]	sgRNA promoter[b]	*B. burgdorferi* strain number	Phenotype without IPTG[c]	
					Cell morphology	Culture growth
All-in-one CRISPRi SV[d]	*flaB*	None	P_{syn}	CJW_Bb313	-[f]	-
			P_{flaBS}	CJW_Bb312	-	-
			P_{resTL}	CJW_Bb228	-	-
			P_{resTS}	CJW_Bb290	-	-
			P_{0826L}	CJW_Bb234	-	-
			P_{0826S}	CJW_Bb235	-	-
			P_{0026}	CJW_Bb314	-	-
		RBSmut	P_{syn}	CJW_Bb381	-	-
		-10TC	P_{syn}	CJW_Bb385	-	-
	rodA	None	P_{syn}	CJW_Bb346	Bulges[g]	Slow[i]
			P_{0526}	CJW_Bb380	Bulges	Slow
		-10AC1	P_{syn}	CJW_Bb375	Bulges	Slow
		-10AC2	P_{syn}	CJW_Bb376	Bulges	Slow
		-10AC12	P_{syn}	CJW_Bb367	Bulges, mild[h]	Slow
		RBSmut	P_{syn}	CJW_BB368	-	-
		-10TC	P_{syn}	CJW_Bb369	-	-
	mreB	None	P_{syn}	N.O.[e]	N/A	N/A
			P_{0526}	N.O.	N/A	N/A
		RBSmut	P_{syn}	CJW_Bb382	Bulges, mild	-
		-10TC	P_{syn}	CJW_Bb398	Bulges, mild	-
	ftsI	None	P_{syn}	CJW_Bb351	Filamentation, mild	Slow
			P_{0526}	CJW_Bb363	Filamentation, mild	Slow
		RBSmut	P_{syn}	CJW_Bb383	-	-
		-10TC	P_{syn}	CJW_Bb386	-	-
Chromosomal *dcas9*	*flaB*	None	P_{syn}	CJW_BB404	-	-
	rodA	None	P_{syn}	CJW_Bb408	-	-
	mreB	None	P_{syn}	CJW_Bb407	Bulges, mild	-
	ftsI	None	P_{syn}	CJW_Bb406	-	-

[a] See Fig. 1H and S1F;
[b] See Table S1;
[c] Phenotypes listed reflect our observations during strain generation or clone expansion in liquid medium. Quantification was not performed;
[d] SV, shuttle vector;
[e] N.O., not obtained, likely due to lethality associated with high depletion level of MreB;
[f] No obvious morphology or growth defect observed.
[g] Cell bulges observed primarily by darkfield microscopy;
[h] Mild phenotype indicates that only a few cells in the population could be observed to have the noted phenotype;
[i] Slow growth indicates either a prolonged time needed to obtain colonies in semisolid BSK-agarose medium or a slower increase in density of liquid cultures as observed by darkfield imaging.

Figure S1

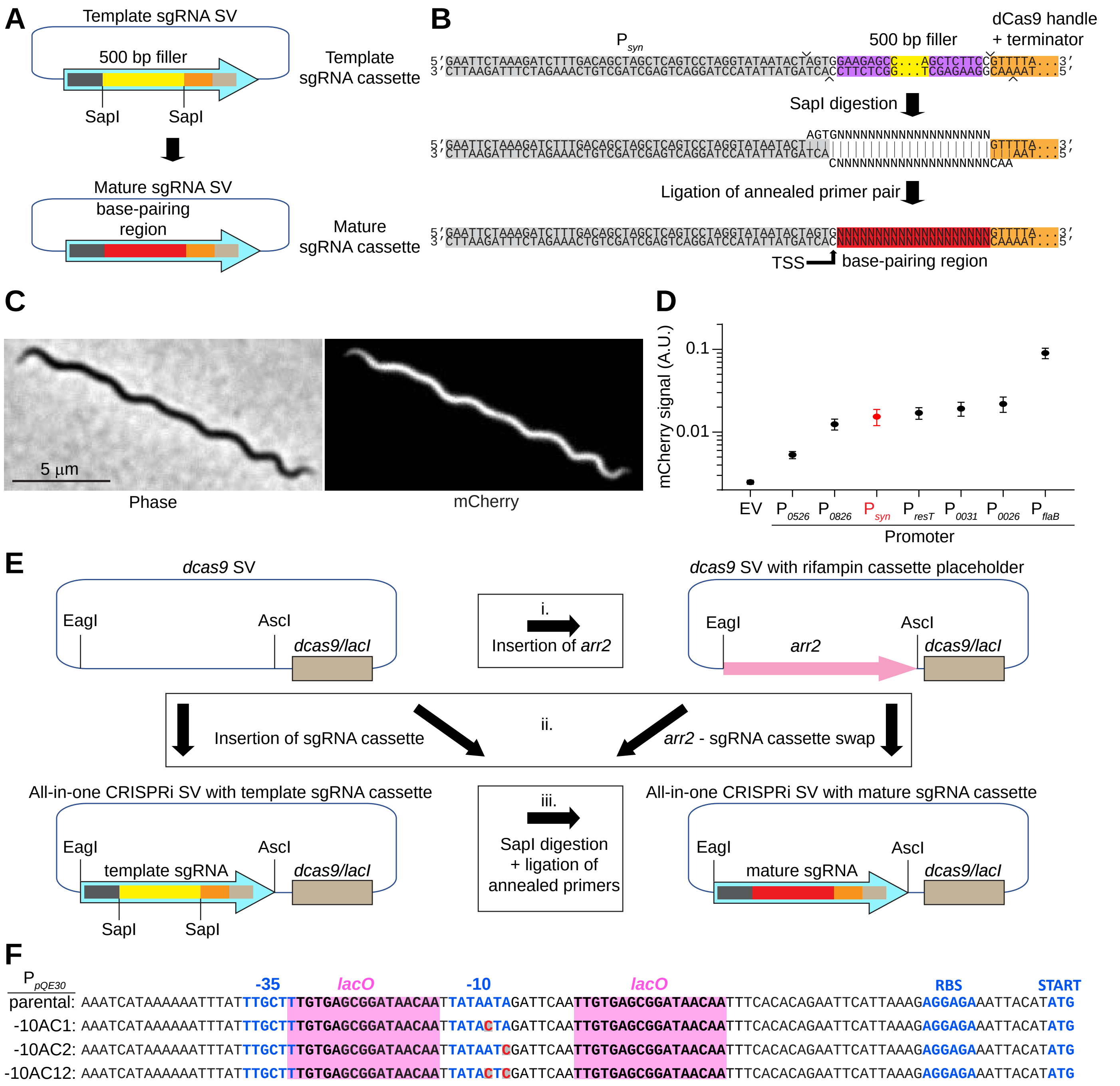

Figure S1. CRISPRi platform construction details.
A. Schematic of conversion of a template sgRNA shuttle vector (SV, top) into a mature sgRNA cassette shuttle vector (bottom).
B. Detail of the process outlined in A. The promoter shown is P_{syn}. The 500 base-pair (bp) DNA filler is released by SapI digestion. Annealed primers are then ligated to the resulting backbone, generating the mature sgRNA's base-pairing region placed immediately downstream of the promoter's transcriptional start site (TSS).
C. Phase-contrast and fluorescence images of a cell of strain CJW_Bb122 expressing mCherry from P_{syn}.
D. Quantification of the P_{syn} strength through cellular mCherry fluorescence intensity measurements. The data for all other promoters was obtained and presented in reference (10). A.U., arbitrary units; EV, empty vector.
E. Cloning avenues for generation of all-in-one CRISPRi shuttle vectors. Top left: *dcas9* shuttle vector. Insertion (cloning path i.) of a rifampin resistance cassette, *arr2*, between its AscI and EagI sites generates a *dcas9* shuttle vector with a rifampin cassette placeholder (top right). Insertion of a sgRNA cassette between the same sites of the *dcas9* shuttle vector (cloning path ii.) yields all-in-one shuttle vectors carrying either a template sgRNA cassette (bottom left) or a mature sgRNA cassette (bottom right). Using the *dcas9* shuttle vector with a rifampin cassette (top right) as a starting point in this cloning step offers the advantage of easy screening of clones by checking for loss or rifampin resistance. Conversion of the template sgRNA cassette of the all-in-one CRISPRi shuttle vector (bottom left) into the mature sgRNA cassette (bottom right) requires SapI digestion followed by ligation of annealed primers (cloning path iii.), as outlined in panel B.
F. Mutations, other than those shown in Fig. 1H, introduced into the P_{pQE30} sequence in an attempt to decrease basal expression of *dcas9*. *lacO*, LacI binding sites; RBS, ribosome-binding site.

Figure S2

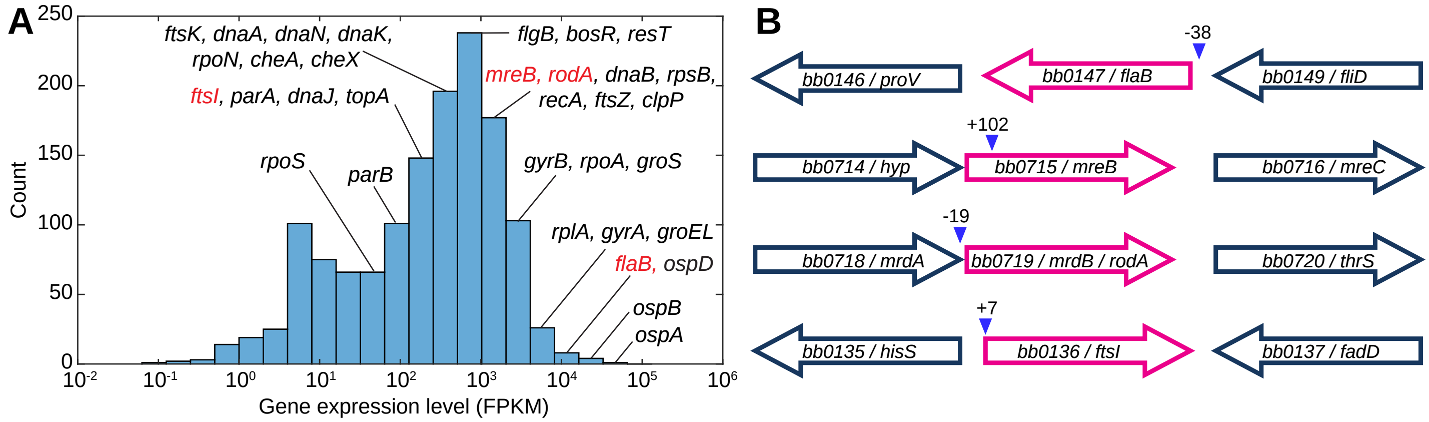

Figure S2. Genes targeted by CRISPRi in *B. burgdorferi.*

A. Histogram showing the distribution of *B. burgdorferi* gene expression levels. The RNAseq data was obtained and reported by Arnold el al. (9) using strain B31-A3 (11) grown in BSK-II liquid culture. The data shown is from the early exponential condition as described in the original publication. Each bin represents a 2-fold range of expression levels. Indicated are expression bins where many physiologically important genes are located. Highlighted in red are the genes targeted by CRISPRi in our study. FPKM, fragments per kilobase per million reads.

B. Genomic context of genes targeted by CRISPRi and location of CRISPR target sites. Coding regions of targeted genes are shown in pink, while upstream and downstream genes are in dark blue. When the distance between two adjacent genes is so short as to suggest that the genes form an operon, the genes are drawn close to each other. However, previous studies (7-9) have identified transcriptional start sites immediately upstream of the START codons of each of these genes. Labels contain both the gene number and its abbreviation. Blue arrowheads show the location of the CRISPR target sites, always within the 5' UTR or the coding sequence of the gene. Values indicate the nucleotide position relative to the translational start site where the 5' foremost complementary base of the sgRNA binds. Arrows are not drawn to scale.

Figure S3

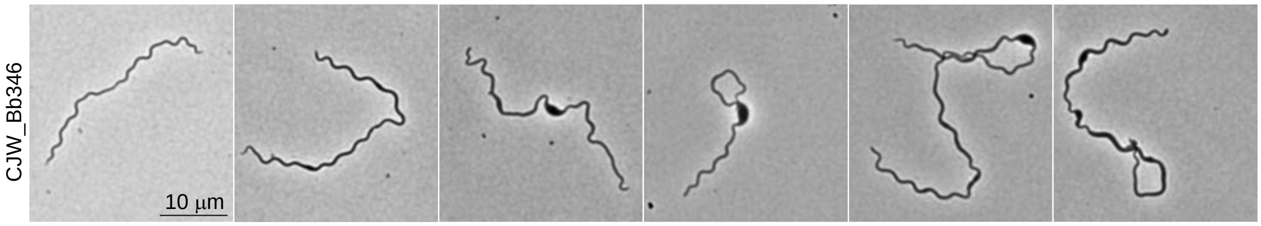

Figure S3. RodA depletion phenotype in the absence of IPTG induction.
Phase contrast images of cells of strain CJW_Bb346 grown without IPTG. A cell with a normal width is at the left, while the other panels show various degrees of cell bulging.

Figure S4

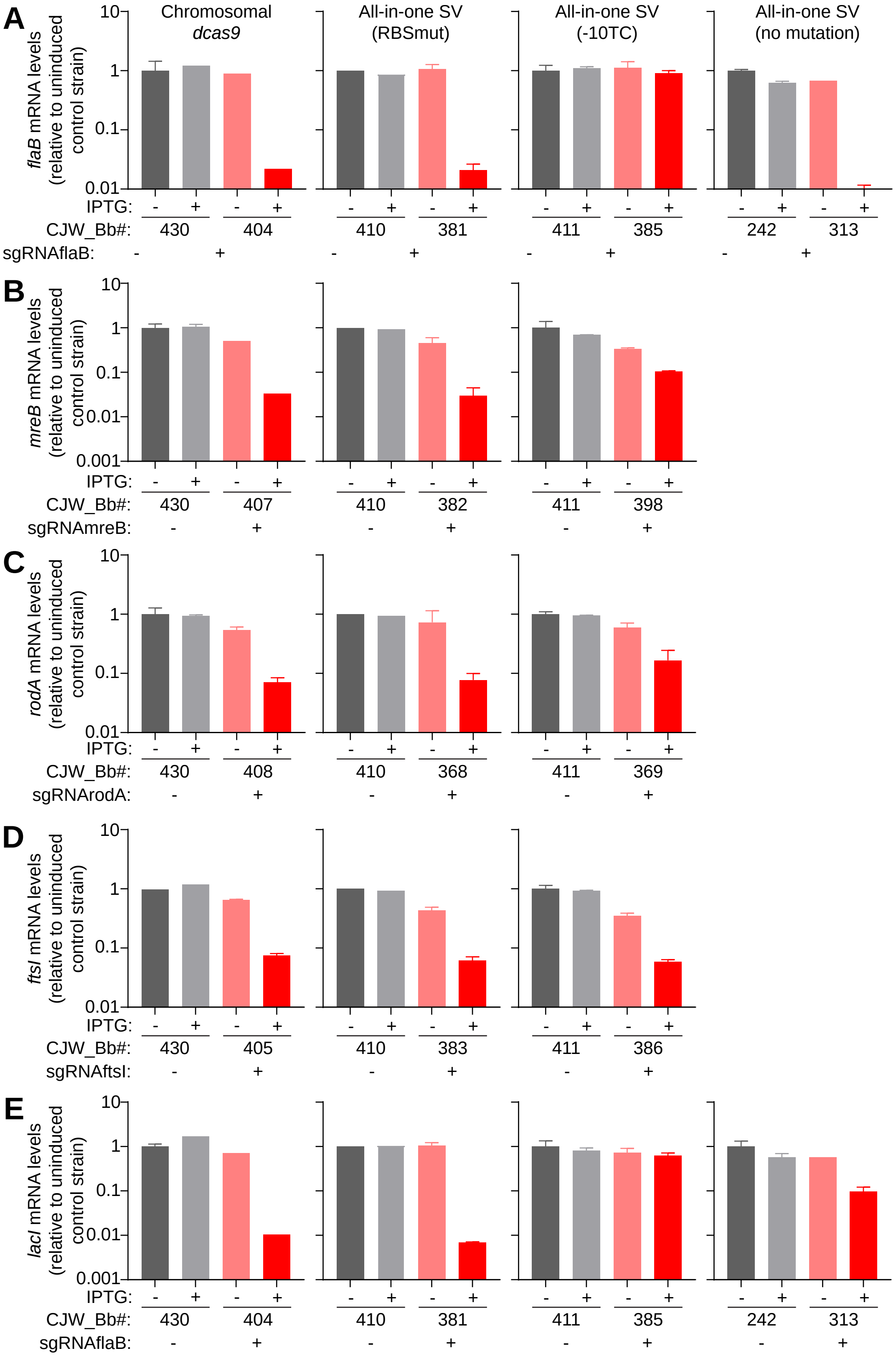

Figure S4. Effect of CRISPRi on targeted gene mRNA levels.
A. *flaB*, **B.** *mreB*, **C.** *rodA*, **D.** *ftsI*, and **E.** *lacI* mRNA levels measured in the indicated control strains (gray) and CRISPRi depletion strains (pink and red) after two days of growth with or without IPTG. Shown are the means ± standard deviations measured from two cultures, or the values of single measurements (when no error bar is present). The version of the CRISPRi platform carried by each set of strains is indicated above the corresponding column of graphs. SV, shuttle vector.

Figure S5

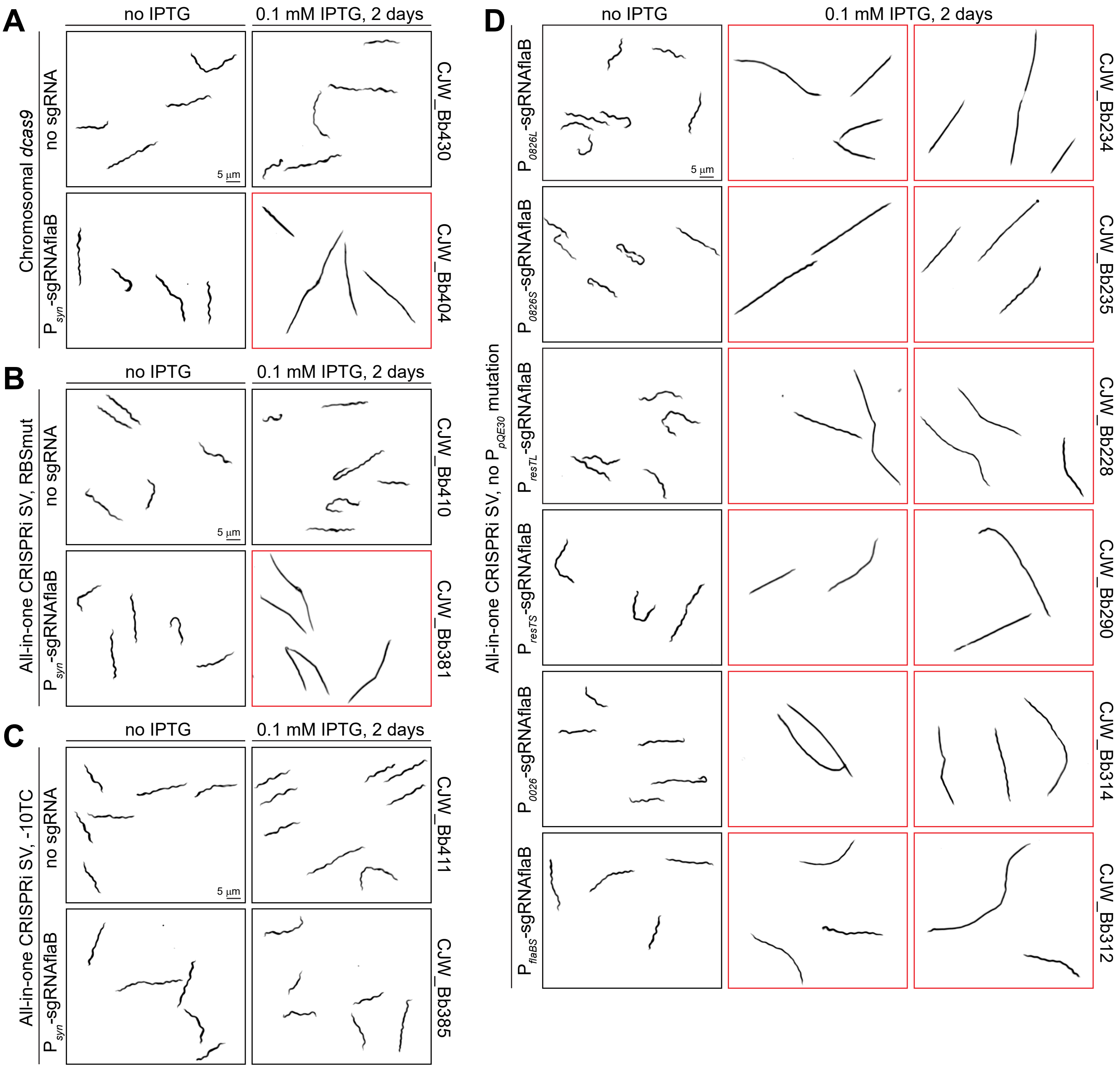

Figure S5. Darkfield microscopy characterization of flagellin depletion strains.
A-C. Inverted darkfield images of strains expressing either no sgRNA or sgRNAflaB from versions of the CRISPRi platform that reduce the basal expression of *dcas9*.
D. Inverted darkfield images of strains carrying *flaB*-targeting all-in-one CRISPRi shuttle vectors. The strains differ only in the promoter used to drive sgRNAflaB expression, as noted.
A-D. Images showing a flagellin depletion phenotype are outlined in red. SV, shuttle vector.

Figure S6

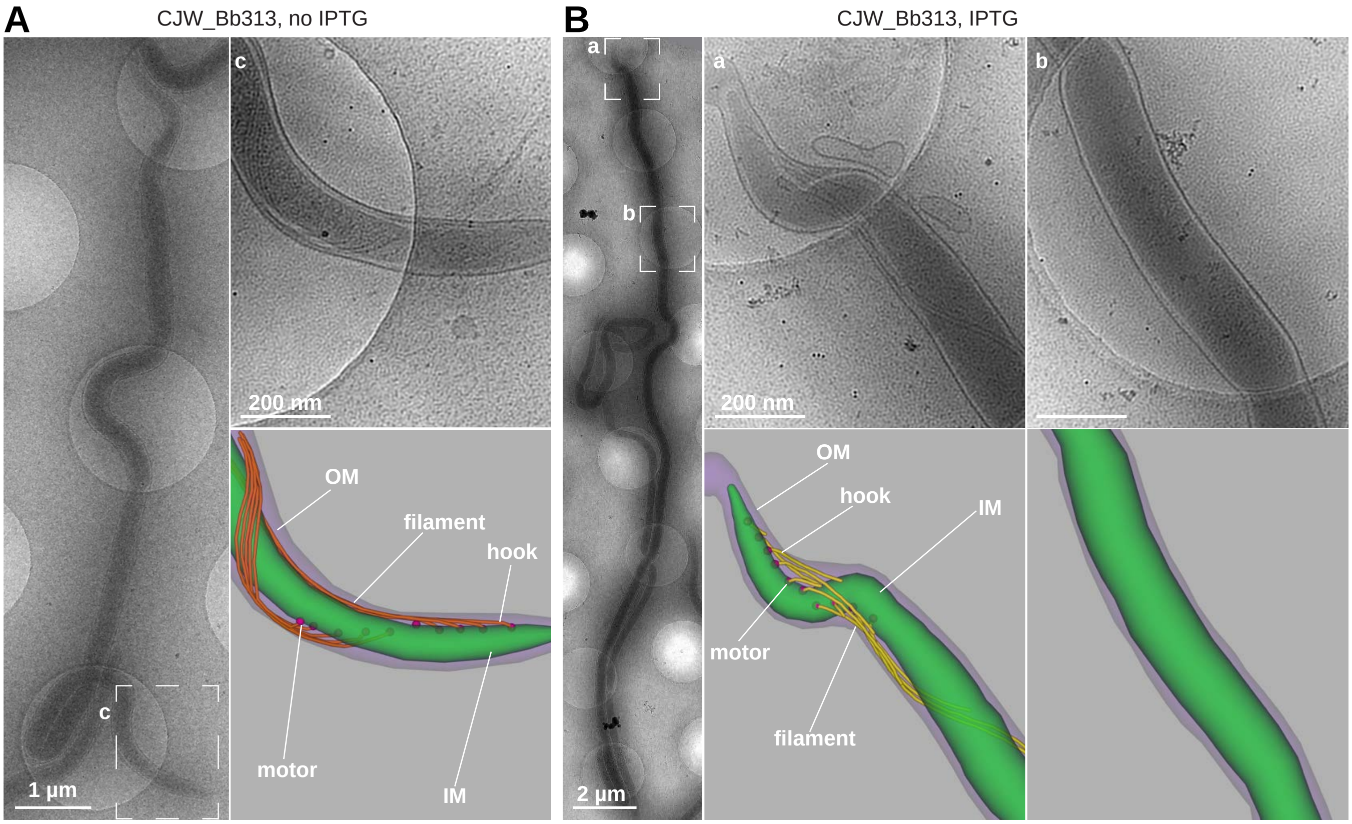

Figure S6. Cryo-ET characterization of flagellin depletion.

A. Cryo-ET-based detection of periplasmic flagella in a cell of strain CJW_Bb313 grown in the absence of IPTG. Shown at the left is a low magnification view of the same cell as in Fig. 4C. Top right: high magnification view of the bottom end (c) of the cell. Bottom right: three-dimensional segmentation of the bottom end region of the cell.

B. Flagellin depletion assessed by cryo-ET in a cell of strain CJW_Bb313 after two days of IPTG exposure. Left: low magnification view of the entire cell. Top center and right: high magnification views of the end (a) and center (b) of the cell, respectively. Bottom center and right: three-dimensional segmentation of the end and center of the cell, respectively.

Figure S7

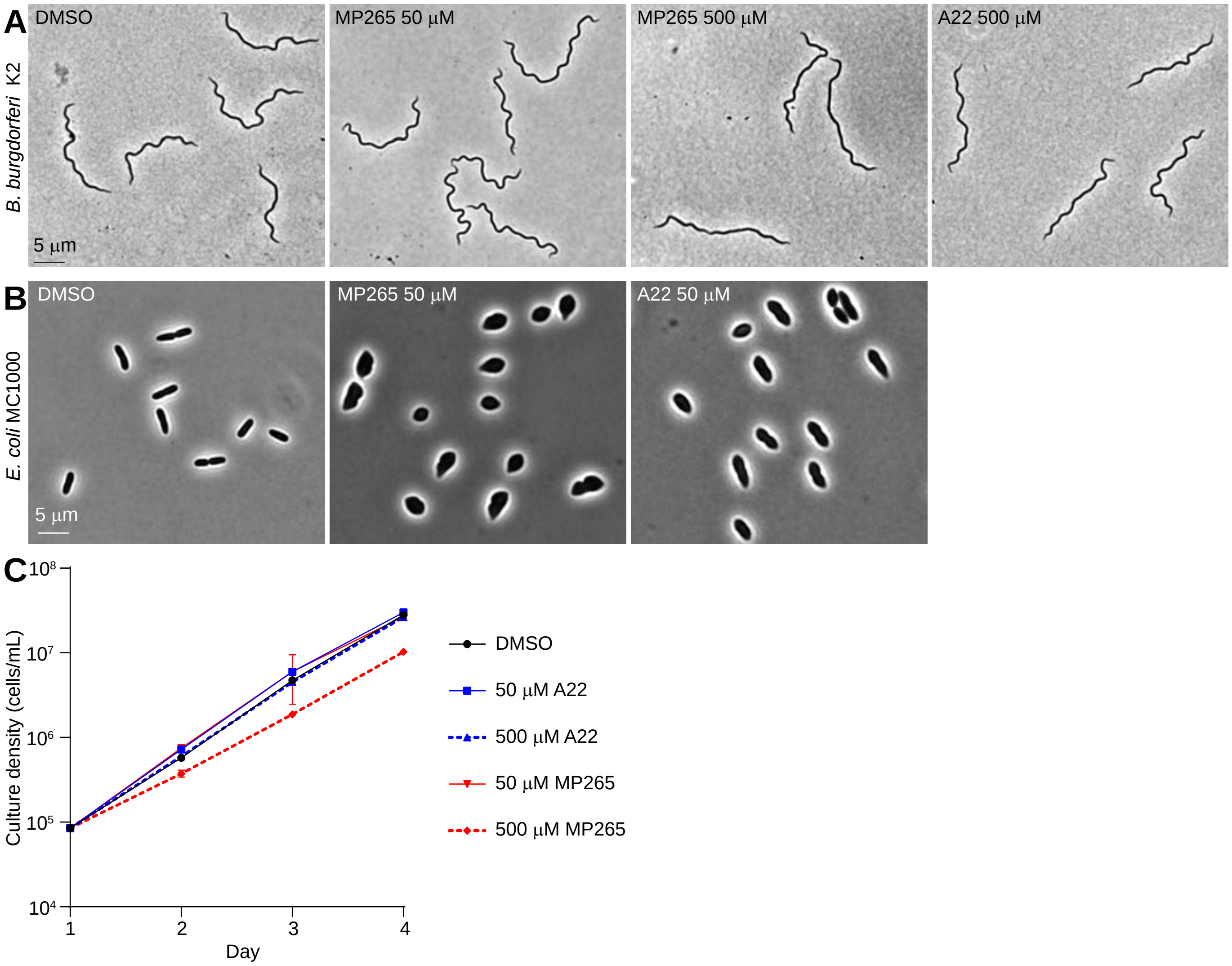

Figure S7. Phenotypic characterization of *B. burgdorferi* following exposure to MreB inhibitors.
A. Phase contrast images of cells of *B. burgdorferi* strain K2 grown for two days in the presence of 0.1% DMSO or the indicated concentrations of MP265 or A22.
B. Phase contrast images of *E. coli* strain MC1000 grown for one hour in BSK-II medium supplemented with DMSO, MP265, or A22.
C. Growth curve of strain K2 treated with DMSO, MP265, or A22. Two replicate cultures were counted daily for each condition. Shown are means ± standard deviations.

SUPPLEMENTAL MOVIE LEGENDS

All movies were recorded by imaging live *B. burgdorferi* cells in BSK-H medium using darkfield microscopy. Stream acquisition with an exposure time of 200 ms was used. Shown are inverted images. Elapsed time (in seconds) is displayed in the top right corner. A 10-μm scale bar is in the bottom right corner.

Movie_S1. A cell of strain CJW_Bb242 (control strain that carries a *dcas9* shuttle vector but does not express a sgRNA) grown in the absence of IPTG.

Movie_S2. Cells of strain CJW_Bb242 grown in the presence of 0.1 mM IPTG for two days.

Movie_S3. Cells of strain CJW_Bb313 (FlaB depletion strain carrying an all-in-one CRISPRi shuttle vector targeting *flaB* and the unmutated P_{pQE30} promoter controlling *dcas9* expression) grown in the absence of IPTG.

Movie_S4. A cell of strain CJW_Bb313 grown with 0.1 mM IPTG for two days.

Movie_S5. Cell of strain CJW_Bb313 grown with 0.1 mM IPTG for two days.

Movie_S6. Cell of strain CJW_Bb313 grown with 0.1 mM IPTG for two days.

Movie_S7. Cells of strain CJW_Bb313 grown with 0.1 mM IPTG for two days.

Movie_S8. A cell of strain CJW_Bb313 grown with 0.1 mM IPTG for two days. The cell appears almost perfectly straight, like cells of a Δ*flaB* strain (12). The kink at the middle represents the division site.

SUPPLEMENTAL REFERENCES

1. **Gitai Z, Dye NA, Reisenauer A, Wachi M, Shapiro L.** 2005. MreB actin-mediated segregation of a specific region of a bacterial chromosome. Cell **120:**329-341.
2. **Takacs CN, Poggio S, Charbon G, Pucheault M, Vollmer W, Jacobs-Wagner C.** 2010. MreB drives de novo rod morphogenesis in *Caulobacter crescentus* via remodeling of the cell wall. J Bacteriol **192:**1671-1684.
3. **Iwai N, Nagai K, Wachi M.** 2002. Novel S-benzylisothiourea compound that induces spherical cells in *Escherichia coli* probably by acting on a rod-shape-determining protein(s) other than penicillin-binding protein 2. Biosci Biotechnol Biochem **66:**2658-2662.
4. **Iwai N, Ebata T, Nagura H, Kitazume T, Nagai K, Wachi M.** 2004. Structure-activity relationship of S-benzylisothiourea derivatives to induce spherical cells in *Escherichia coli*. Biosci Biotechnol Biochem **68:**2265-2269.
5. **Iwai N, Fujii T, Nagura H, Wachi M, Kitazume T.** 2007. Structure-activity relationship study of the bacterial actin-like protein MreB inhibitors: effects of substitution of benzyl group in S-benzylisothiourea. Biosci Biotechnol Biochem **71:**246-248.
6. **Slamti L, de Pedro MA, Guichet E, Picardeau M.** 2011. Deciphering morphological determinants of the helix-shaped *Leptospira*. J Bacteriol **193:**6266-6275.
7. **Bono JL, Elias AF, Kupko JJ, 3rd, Stevenson B, Tilly K, Rosa P.** 2000. Efficient targeted mutagenesis in *Borrelia burgdorferi*. J Bacteriol **182:**2445-2452.
8. **Adams PP, Flores Avile C, Popitsch N, Bilusic I, Schroeder R, Lybecker M, Jewett MW.** 2017. In vivo expression technology and 5' end mapping of the *Borrelia burgdorferi* transcriptome identify novel RNAs expressed during mammalian infection. Nucleic Acids Res **45:**775-792.
9. **Arnold WK, Savage CR, Brissette CA, Seshu J, Livny J, Stevenson B.** 2016. RNA-seq of *Borrelia burgdorferi* in multiple phases of growth reveals insights into the dynamics of gene expression, transcriptome architecture, and noncoding RNAs. PLoS One **11:**e0164165.

10. **Takacs CN, Kloos ZA, Scott M, Rosa PA, Jacobs-Wagner C.** 2018. Fluorescent proteins, promoters, and selectable markers for applications in the Lyme disease spirochete *Borrelia burgdorferi*. Appl Environ Microbiol **84:**e01824-18.

11. **Elias AF, Stewart PE, Grimm D, Caimano MJ, Eggers CH, Tilly K, Bono JL, Akins DR, Radolf JD, Schwan TG, Rosa P.** 2002. Clonal polymorphism of *Borrelia burgdorferi* strain B31 MI: implications for mutagenesis in an infectious strain background. Infect Immun **70:**2139-2150.

12. **Motaleb MA, Corum L, Bono JL, Elias AF, Rosa P, Samuels DS, Charon NW.** 2000. *Borrelia burgdorferi* periplasmic flagella have both skeletal and motility functions. Proc Natl Acad Sci U S A **97:**10899-10904.
